# Rapid‐Turnaround Co‐Administration of mRNA‐Based MHC‐I and MHC‐II‐Restricted Neoantigens Enhances Immune Responses of Antigen‐Specific CD8^+^ T Cells and Anti‐Cancer Efficacy in Colorectal Cancer

**DOI:** 10.1002/advs.202506426

**Published:** 2025-07-23

**Authors:** Seongje Cho, Woori Kwak, Hyunho Yoon, Jisun Lee, Seonghyun Lee, Hyo‐Jung Park, Sohee Jo, Yu‐Sun Lee, Yu‐Jeong Seo, Youngran Cho, Seo‐Hyeon Bae, Subin Yoon, Gahyun Roh, Dahyeon Ha, Ayoung Oh, Eun‐Jin Choi, Soo‐Yeon Lee, Huijeong Choi, Jungmin Kim, Yeeun Lee, Sowon Lee, Sang‐In Park, Dae‐Keun Kim, Jun Chang, Ki Tae Kim, Kwoneel Kim, Jae‐Hwan Nam

**Affiliations:** ^1^ Department of Medical and Biological Sciences The Catholic University of Korea Bucheon Gyeonggi‐do 14662 Republic of Korea; ^2^ BK Four Department of Biotechnology The Catholic University of Korea Bucheon Gyeonggi‐do 14662 Republic of Korea; ^3^ Graduate School of Pharmaceutical Sciences Ewha Womans University Seoul 03760 Republic of Korea; ^4^ Department of Biomedical Laboratory Science Daegu Haany University Gyeongsan 38578 Republic of Korea; ^5^ SML Biopharm Gwangmyeong 14303 Republic of Korea; ^6^ Department of Molecular Genetics & Dental Pharmacology School of Dentistry Seoul National University Seoul 03080 Republic of Korea; ^7^ Dental Research Institute and Dental Multi‐omics Center Seoul National University Seoul 03080 Republic of Korea; ^8^ Department of Biomedical and Pharmaceutical Sciences Kyung Hee University Seoul 02447 Republic of Korea; ^9^ Department of Biology Kyung Hee University Seoul 02447 Republic of Korea

**Keywords:** colorectal cancer, lipid nanoparticle, mRNA, neoantigen, personalized cancer vaccine, post‐surgery recurrence

## Abstract

Personalized cancer vaccines (PCVs) represent a promising frontier in cancer immunotherapy; however, challenges in neoantigen prediction and treatment optimization persist. This study aims to introduce an innovative mRNA‐based PCV platform that addresses these limitations. Co‐administration of our major histocompatibility complex (MHC)‐I and MHC‐II‐restricted neoantigens increases antigen‐specific T cell responses and exhibits strong anti‐cancer efficacy, significantly inducing antigen‐specific CD8^+^ T cell‐immune responses. The mRNA‐based vaccine targeting the novel antigens demonstrates anti‐tumor efficacy in a murine model of colorectal cancer and reduces post‐surgery recurrence in mRNA‐vaccinated mice. Notably, early‐stage vaccination induces a striking anti‐cancer effect, underscoring the critical role of MHC‐II neoantigens alongside MHC‐I antigen prediction in shaping effective anti‐tumor immunity, in the activation of antigen‐specific CD8^+^ T cells. In addition, the combination of immune checkpoint inhibitors and the vaccine synergistically inhibits tumor growth by inducing robust T cell responses and promoting favorable alterations in the tumor microenvironment. Taken together, the results provide a strong rationale for the clinical investigation of rapid‐turnaround co‐administration of MHC‐I/II‐restricted neoantigens‐based mRNA vaccines in colorectal cancer, as supported by the anti‐tumor efficacy of early‐stage application and combination immunotherapy approaches.

## Graphical abstract

Co‐administration with MHC class I‐ and II‐restricted neoantigens encoding mRNA‐based personalized cancer vaccines in a mouse CRC model can induce antigen‐specific immune responses and anti‐cancer effects. Notably, vaccination at an early stage of cancer development can have potent anti‐cancer effects. Furthermore, combining mRNA with immune checkpoint inhibitors synergistically inhibits tumor growth.

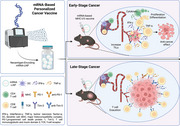

## 1 Introduction

Colorectal cancer is the second leading cause of death worldwide, accounting for 8% of all cancer deaths.^[^ ^1^ ^]^ Early‐stage colorectal cancer can be treated surgically; however, recurrence is common, and drug resistance develops,^[^ ^2^ ^]^ underscoring the urgent need for effective treatments. Recent studies have explored immunotherapy approaches to prevent recurrence and metastasis in colorectal cancer treatment.^[^ ^3^ ^]^ Cancer immunotherapy is a groundbreaking approach that harnesses the power of the immune system to effectively combat malignant cells.^[^ ^4^ ^]^ Personalized cancer vaccines (PCVs) can elicit specific anti‐tumor immune responses by targeting patient‐specific neoantigens.^[^ ^5^ ^]^ Neoantigens, mainly arising from somatic mutations in cancer, exist only in cancer tissues, making them ideal targets for immunotherapy.^[^ ^6^ ^]^ Advances in next‐generation sequencing (NGS), bioinformatics, and machine learning have significantly facilitated neoantigen identification, driving the development of PCVs.^[^ ^7^ ^]^ Unlike traditional anti‐cancer medications, PCVs leverage the patient's immune system to induce anti‐cancer effects, minimizing adverse reactions while triggering immune responses tailored to the unique neoantigens found exclusively in cancer cells.^[^ ^8^ ^]^

Messenger ribonucleic acid (mRNA) technology has emerged as a versatile platform for delivering neoantigens and inducing robust T cell responses.^[^ ^9^ ^]^ Recently, mRNA vaccines offer several advantages, including rapid production, the absence of genomic integration risk, and the ability to encode multiple neoantigens.^[^ ^10^ ^]^ Regarding stimulating T cell responses, mRNA vaccines have shown superior efficacy compared to protein‐based vaccines.^[^ ^11^ ^]^ Clinical trials on mRNA‐based PCVs in pancreatic cancer and melanoma have yielded encouraging results, highlighting their significant potential.^[^ ^12^ ^]^ Given these observations, an important aspect of enhancing the efficacy of mRNA cancer vaccines is selecting neoantigens based on their binding affinity to major histocompatibility complex (MHC) molecules. Recent research has predominantly focused on MHC‐I‐restricted neoantigens to elicit CD8^+^ T cell responses; however, the significance of MHC‐II‐restricted neoantigens and CD4^+^ T cell responses in anti‐tumor immunity warrants further investigation.^[^ ^13^ ^]^ Furthermore, determining the ideal timing of cancer vaccination and its combination with immune checkpoint inhibitors (ICIs) relative to tumor progression could significantly influence therapeutic efficacy.^[^ ^14^ ^]^

In this study, neoantigens were identified from somatic mutations using NGS. The immunogenicity of mRNA cancer vaccines and their anti‐tumor efficacy—encoding neoantigens with MHC‐I or MHC‐II binding capabilities—were investigated in a murine colon cancer model. Additionally, the optimal timing for cancer vaccination, based on tumor progression, and its consequent impact on therapeutic outcomes were determined. This study provides valuable insights into the design and application of mRNA‐based PCVs, highlighting the importance of increasing CD8^+^ T cell responses through co‐administration of MHC‐I and MHC‐II neoantigens, early intervention, and combination treatment with ICIs in colon cancer immunotherapy.

## 2 Results

### 2.1 Neoantigen Prediction

**Figure 1:**
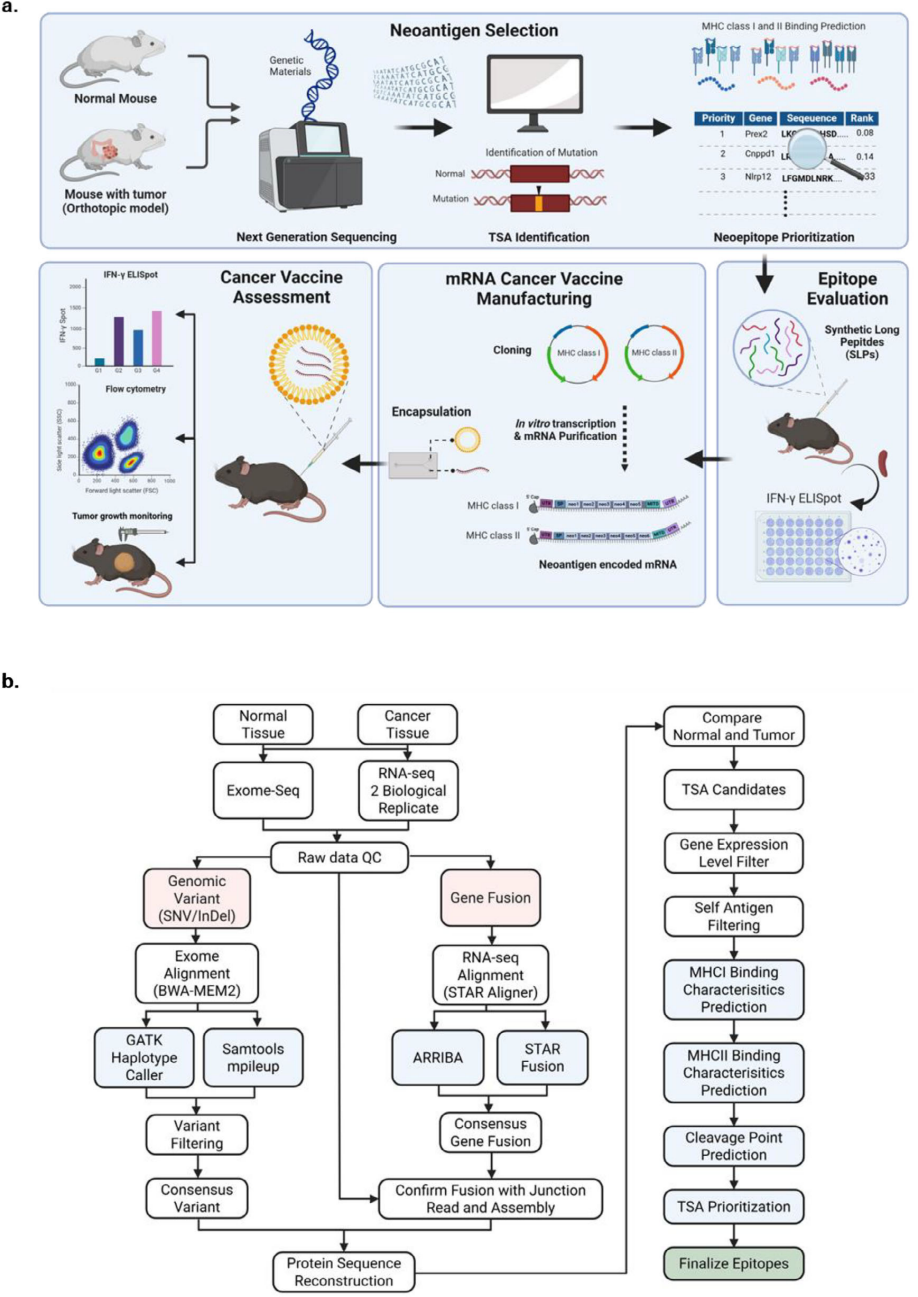
Schematic illustration of neoantigen validation. a) Genes of normal mice and tumor‐bearing orthotopic cancer mouse models were analyzed to identify mutations. Subsequently, neoantigen candidates were prioritized based on whole exome sequencing (WES), expression levels, and major histocompatibility complex (MHC) binding ability. The immunogenicity of neoantigens was evaluated using synthetic long peptides. Immunogenic neoantigens encoded in mRNA‐lipid nanoparticles (LNPs) were administered to tumor‐bearing mice to assess their potential as neoantigen‐based cancer vaccines. b)The workflow for neoantigens derived from the candidate variants and gene fusions. Graphics created using BioRender (www.BioRender.com).

In conventional PCV neoantigen design, epitopes are typically crafted by targeting somatic mutations uniquely present in cancer tissues. This study employed an orthotopic model using the MC38 cell line to leverage the distinct genomic differences for inducing tumor‐specific immune responses (**Figure** **1**a). Specifically, germline mutations inherent to the mouse strain and cell line used for neoantigen design were targeted. Table S1, Supporting Information details the number of variants identified by the two variant callers, along with those obtained after filtering. Variants exclusive to the tumor were identified by employing two variant callers, resulting in 546 single nucleotide variants (SNVs) and 68 insertions/deletions (InDels) which were selected as consensus candidates for neoantigen design.

ARRIBA and STAR fusion were used to analyze gene fusions in two mRNA‐seq datasets from normal and tumor samples, respectively. This comparison revealed tumor‐specific gene fusions. ARRIBA identified 18 tumor‐specific gene fusions, whereas STAR fusion identified 12, with 8 fusions common to both tools. Further filtering using *de novo* assembly with Trinity and read support at fusion junctions excluded two gene fusions (*Eefsec_Mcm2*, *Smurf1_Kpna7*) due to insufficient junction reads. Consequently, six gene fusions were shortlisted as candidates for neoantigen design.

Following the workflow in Figure 1b, neoantigens derived from the candidate variants and gene fusions were prioritized. The prioritization process considered MHC binding rankings, the expression levels of the respective mutations, and their uniqueness compared with self‐antigens. Therefore, 20 MHC‐I and 12 MHC‐II‐restricted neoantigens were designed and selected, respectively. Tables S2 and S3 (Supporting Information) present a comprehensive list of designed epitopes, along with their target sources and associated genes.

### 2.2 Immunogenicity of Predicted Neoantigens

**Figure 2:**
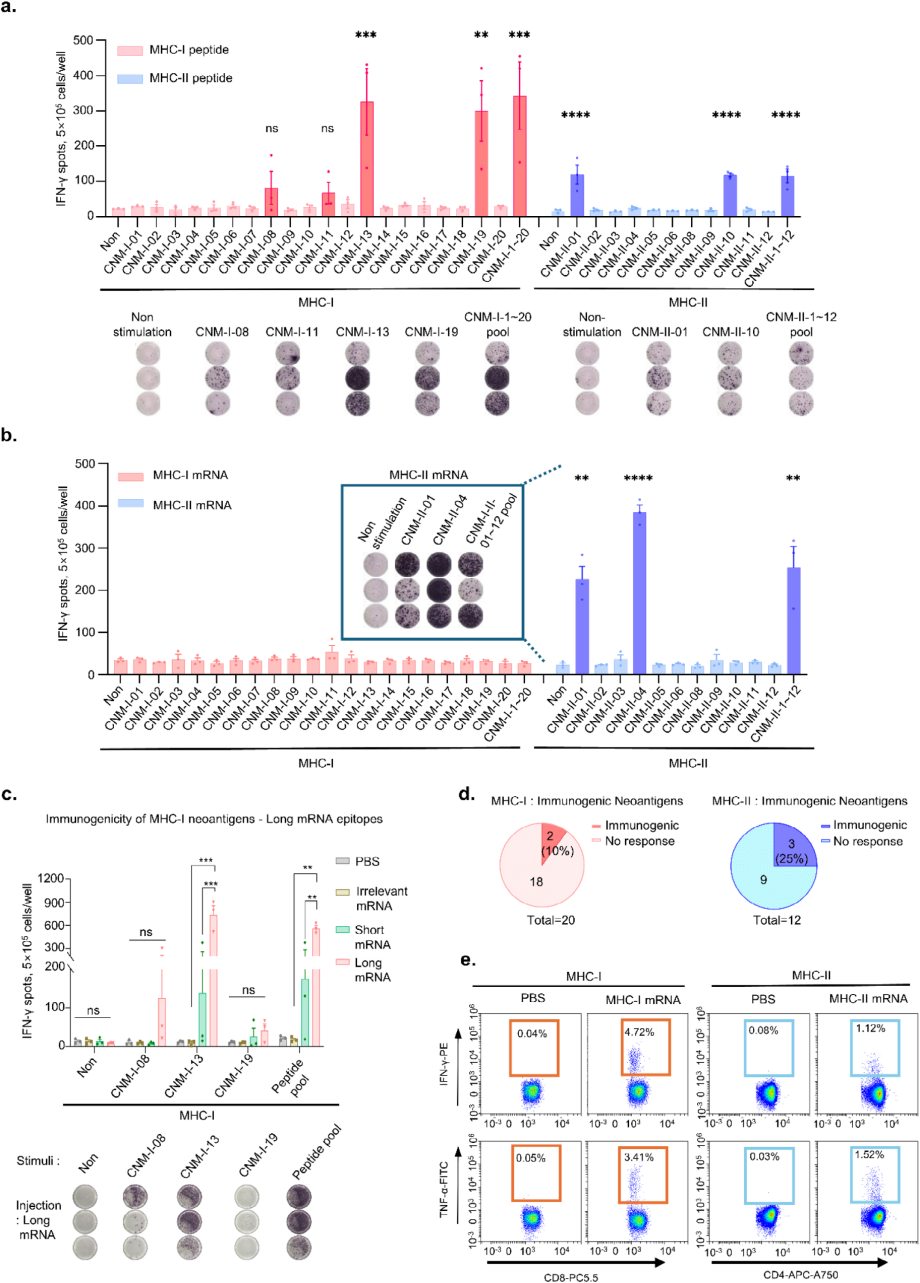
Immunogenicity identification of predicted neoantigen candidates. a) Immunogenicity of peptide‐based neoantigens. C57BL/6J mice (n = 3 per group) were immunized with MHC‐I or MHC‐II peptides (100 µg) mixed with poly I:C (50 µg). Seven days after the last immunization, IFN‐γ ELISpot analysis was performed on splenocytes stimulated with the indicated peptides. b) Immunogenicity of mRNA‐based neoantigens. Mice were immunized with 20 µg of mRNA‐lipid particles (LNPs) encoding each sequence of MHC‐I or MHC‐II neoantigens (n = 3 per group). c) IFN‐γ ELISpot analysis comparing immunogenicity based on antigen length of mRNA vaccines. C57BL/6J mice (n = 3 per group) were immunized with MHC‐I antigens (CNM‐I‐08, CNM‐I‐13, and CNM‐I‐19) as short mRNA (8‐ to 10‐mer) and long mRNA (27‐mer). The splenocytes were stimulated with each neoantigen. d) Immunogenic prevalence of MHC class‐I or class‐II restricted neoantigens. e. Representative data of cytokine secretion by neoantigens based on the MHC pathway. Symbols in the graphs represent a single mouse with mean ± standard error of the mean (SEM), and significance was determined using one‐way ANOVA and unpaired two‐tailed Student's *t*‐test (***p < 0.001, **p < 0.01, *p < 0.05).

These neoantigen candidates were synthesized as synthetic long peptides (SLPs) to evaluate the immunogenicity of candidate neoantigens from a pool of 32 candidates with high prediction scores for MHC‐I and MHC‐II binding. The 20 neoantigens associated with MHC‐I were synthesized as 8–10 amino acid (aa) peptides, whereas the 12 neoantigens associated with MHC‐II were synthesized as 27 aa peptides. These peptides were pooled, mixed with poly I:C, and administered subcutaneously to C57BL/6 mice once weekly. Splenocytes were harvested and subjected to interferon‐gamma (IFN‐γ) ELISpot analysis 1 week after the last immunization (Figure S1, Supporting Information). Two antigens (CNM‐I‐13 and CNM‐I‐19) in MHC‐I‐associated neoantigens exhibited robust antigen‐specific IFN‐γ secretion (**Figure** **2**a). Two MHC‐II‐associated neoantigens (CNM‐II‐01 and CNM‐II‐10) exhibited significant antigen‐specific IFN‐γ secretion (Figure 2a).

Subsequently, mRNA encoding the corresponding neoantigens were constructed and characterized (Figure S2a,b, Supporting Information). Each mRNA was encapsulated in lipid nanoparticles (LNPs) and the mRNA‐LNPs were analyzed by particle size, polydispersity index (PDI), and encapsulation efficiency (Figure S2c–e, Supporting Information). The characterized mRNA vaccines were subcutaneously injected into C57BL/6 mice on days 0, 4, 7, and 14. mRNA vaccines encoding MHC‐II‐restricted neoantigens (CNM‐II‐01 and CNM‐II‐04) significantly increased IFN‐γ secretion; however, IFN‐γ induction did not occur during treatment with mRNA vaccines encoding MHC‐I‐associated neoantigens (Figure 2b). Given that neoantigens length can influence immunogenicity,^[^ ^15^ ^]^ long mRNA (27 aa length) vaccines encoding MHC‐I‐associated neoantigens were constructed (Table S4, Supporting Information). The IFN‐γ ELISpot analysis demonstrated that the long mRNA‐encoded neoantigens were more immunogenic than the short mRNA‐encoded neoantigens (Figure 2c). Overall, 10% of the MHC‐I antigens predicted using the in silico method were immunogenic, whereas 25% of the MHC‐II antigens were immunogenic in our study (Figure 2d).

Flow cytometric analysis was performed to confirm that the predicted pathways for MHC‐I and MHC‐II antigens induced cytokine secretion. MHC‐I neoantigens induced CD8^+^ cell‐specific IFN‐γ and tumor necrosis factor‐alpha (TNF‐α) secretion. In contrast, MHC‐II neoantigens prompted CD4^+^ cell‐specific IFN‐γ and TNF‐α secretion (Figure 2e), suggesting that these identified neoantigens effectively activate the respective T cell pathways through MHC‐I and MHC‐II molecules, thereby validating the reliability of these predictions.

### 2.3 The Impact Of Co‐Administration of MHC‐I and MHC‐II Antigen‐Based mRNAs on Neoantigen‐Specific CD8^+^ T Cell‐Mediated Immune Responses

**Figure 3:**
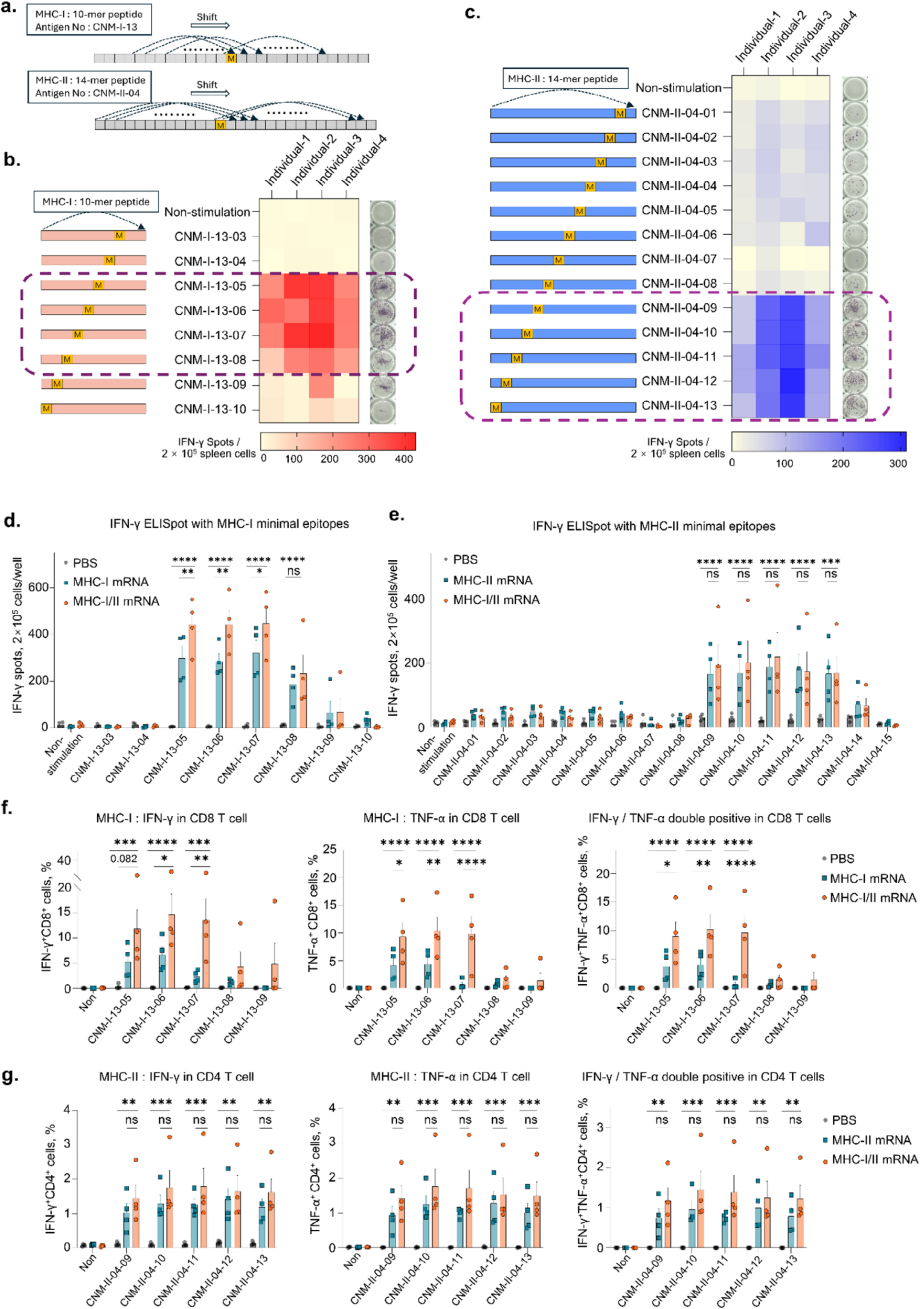
Defining the minimal epitopes for MHC‐I and MHC‐II neoantigens. a) Schematic illustration of peptide design for determination of the minimal epitopes in MHC‐I and MHC‐II neoantigens. b, c) IFN‐γ ELISpot assay results according to stimulation with each minimal epitope after vaccination with mRNA‐based MHC‐I neoantigen b) and MHC‐II neoantigen c). C57BL/6J mice (n = 4 per group) were immunized with PBS, 20 µg of mRNA‐based MHC‐I, MHC‐II neoantigens or co‐administration on 0, 4, 7, and 14 days. Seven days after the last immunization, IFN‐γ ELISpot analysis was performed on splenocytes stimulated with the indicated peptides. d, e) IFN‐γ ELISpot analysis comparing immunogenicity based on vaccination with MHC‐I, MHC‐II neoantigens, and co‐administration. The graph shows that the spleen cells were stimulated with MHC‐I minimal epitopes d) and MHC‐II minimal epitopes e). f g) Secretion of each minimal epitope‐specific cytokines (IFN‐γ and TNF‐α) from CD8^+^ T cells (f) and CD4^+^ T cells g) in spleen cells. Symbols in the graphs represent a single mouse with mean ± standard error of the mean (SEM), and significance was determined using one‐way ANOVA and unpaired two‐tailed Student's *t*‐test (***p < 0.001, **p < 0.01, *p < 0.05).

Generally, the MHC‐I pathway is activated by peptides that are typically 8 to 10 amino acids in length, while the MHC‐II pathway is known to be activated by longer peptides, ranging from 11 to 14 amino acids in length. To determine the minimal epitopes, we designed 10‐mer peptides (CNM‐I‐13‐01 to CNM‐I‐13‐10; 10 peptides) within MHC‐I antigen (CNM‐I‐13) and 14‐mer peptides (CNM‐II‐04‐01 to CNM‐II‐04‐13; 13 peptides) within MHC‐II antigen (CNM‐II‐04), including each mutation (**Figure** **3**a; Table S5, Supporting Information). C57BL/6 mice were immunized with mRNA‐based MHC‐I neoantigens (CNM‐I‐13), MHC‐II neoantigens (CNM‐II‐04), or co‐administration of both at 0, 4, 7, and 14 days. One week after the last immunization, the splenocytes were harvested from immunized mice, stimulated with each peptide, and evaluated for immunogenicity using IFN‐γ ELISpot.

The synthesis of CNM‐I‐13‐01 and CNM‐I‐13‐02 peptides failed, which appears to reflect a limitation of peptide‐based vaccines due to variations in physicochemical properties depending on amino acid sequences.

Some minimal epitopes of MHC‐I and MHC‐II antigens were found to be non‐immunogenic or weakly immunogenic, even if they contained tumor‐specific mutations. Subsequently, CNM‐I‐13‐05, ‐06, ‐07, and ‐08 in MHC‐I restricted antigen were highly immunogenic (Figure 3b) and CNM‐II‐04‐09, ‐10, ‐11, ‐12, and ‐13 in MHC‐II restricted antigens induced antigen‐specific secretion of IFN‐γ in MHC‐II antigen strongly (Figure 3c), suggesting that not all mutant antigens are presented; only certain of peptide complexes with MHC and TCR to function as antigens.

To next confirm antigen‐specific immune responses elicited by individual antigens and their combination, T cell mediated cytokine levels were evaluated using splenocytes isolated from mice vaccinated with MHC‐I/II antigen, or a combination of both. In the IFN‐γ ELISpot assay, antigen‐specific IFN‐γ secretion by CD8^+^ T cells was significantly higher in the MHC‐I/II co‐administration group compared to the MHC‐I‐only vaccination group (Figure 3d), whereas there were no significant differences in IFN‐γ secretion by CD4⁺ T cells between the MHC‐II vaccination group and the MHC‐I/II co‐administration group (Figure 3e). These patterns were also observed in flow cytometric analysis, which showed that co‐administration with MHC‐I and MHC‐II vaccines significantly increased the secretion of IFN‐γ or TNF‐α in CD8^+^ T cells, but not in CD4^+^ T cells (Figure 3f,g; Figure S3, Supporting Information). These results demonstrated that co‐administration of MHC‐I and MHC‐II not only triggers respective antigen‐specific immune responses but also that CD4⁺ T cell activation stimulated by MHC‐II antigens contributes to the activation of antigen‐specific CD8⁺ T cells. Additionally, spleen cells were collected and stained with PE‐H‐2D^b^ tetramer conjugated with CNM‐13‐06 (10‐mer) to detect MHC‐I antigen‐specific T cells using flow cytometry. It showed that there was inter‐individual variation; however, some mRNA‐vaccinated individuals exhibited tetramer binding. (Figure S4a−c, Supporting Information).

### 2.4 Anti‐Cancer Effect of Selected MHC‐I/II Antigen‐Based mRNA Vaccines

**Figure 4:**
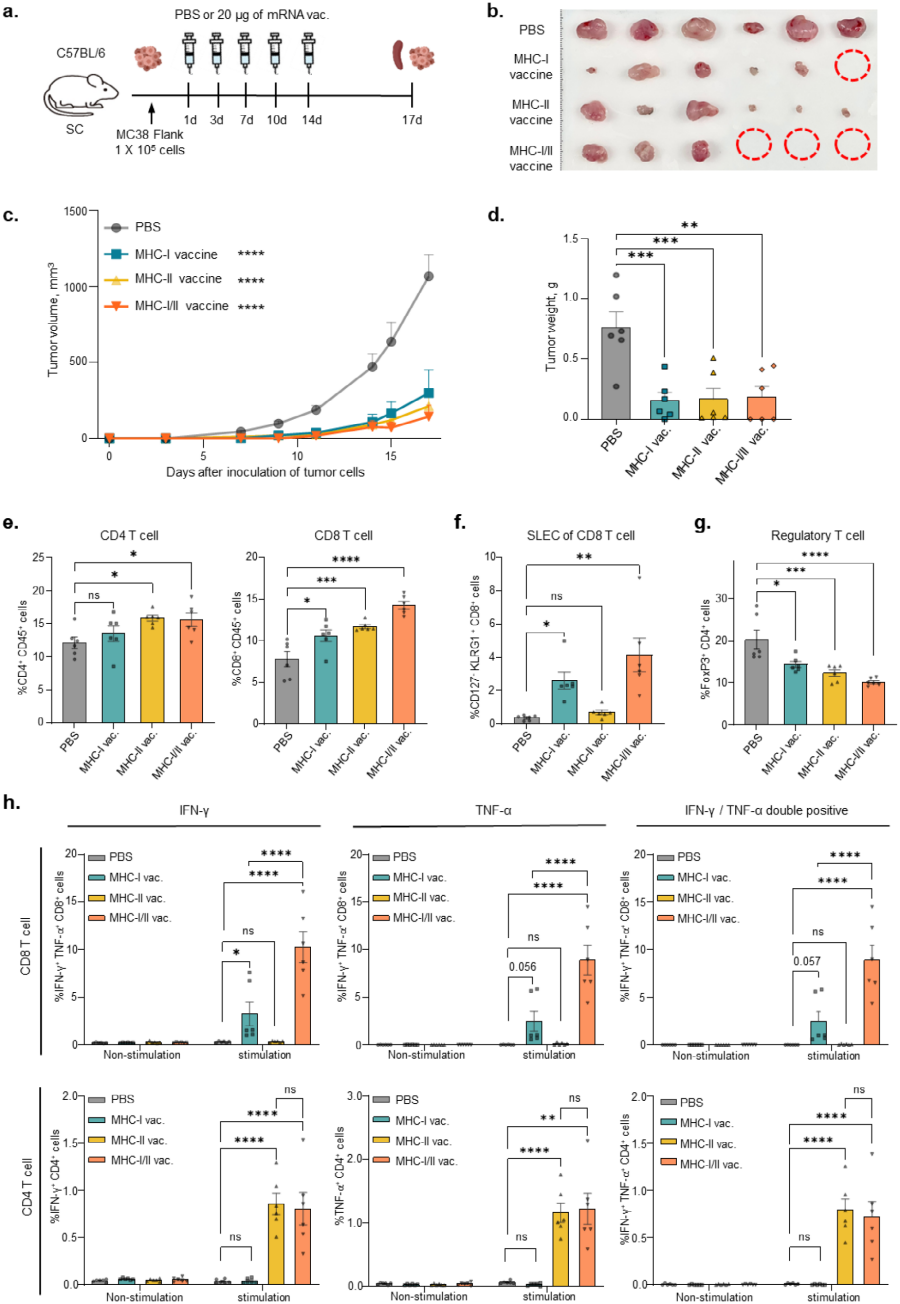
Co‐administration with MHC‐I and MHC‐II‐restricted neoantigens enhanced anti‐cancer efficacy via cell‐mediated immune responses. a) Experimental design. MC38 tumor‐bearing mice (n = 6 per group) were subcutaneously inoculated with MHC‐I, MHC‐II, or both mRNA vaccines (20 µg) on days 1, 3, 7, 10, and 14. b) Tumor images of mRNA vaccine treatment at 17 days after tumor inoculation. c) Tumor growth of the indicated groups (****p < 0.0001, Two‐way ANOVA with Dunnett's multiple comparisons test). d) Weight of tumor tissues. e) Flow cytometric analysis for populations of CD8^+^ T cells (CD8^+^CD45^+^ cells) and CD4^+^ T cells (CD4^+^CD45^+^) in spleen cells. f) Population of short‐lived effector cells (SLECs; KLRG1^+^CD127^‐^) in CD8^+^ cells assessed using flow cytometry. g) Population of regulatory T cells (FoxP3^+^CD4^+^ cells) in spleen cells. h) Secretion of neoantigen‐specific cytokines (IFN‐γ and TNF‐α) from CD8^+^ and CD4^+^ T cells in spleen cells. Data from flow cytometry and ELISpot analysis are shown as mean ± standard error of the mean (SEM), analyzed using one‐way ANOVA with Dunnett's multiple comparisons test and unpaired two‐tailed Student's *t*‐test (****p < 0.0001, ***p < 0.001, **p < 0.01, *p < 0.05).

MC38 tumor‐bearing mice were administered the MHC‐I vaccine, MHC‐II vaccine, or both vaccines to investigate the anti‐cancer efficacy of the mRNA vaccines (**Figure** **4**a). Compared with the phosphate‐buffered saline (PBS) administration group, the mRNA vaccine administration group exhibited a significant decrease in the growth and volume of colon tumors (Figure 4b–d; Figure S5a and S5b, Supporting Information). The group co‐administered the MHC‐I and MHC‐II vaccines exhibited markedly improved the number of tumor‐free mice (3/6) compared to the group administered the MHC‐I (1/6) or MHC‐II vaccine (0/6) alone. Furthermore, spleen cells from vaccinated mice were isolated to analyze the T cell population (Figure S6, Supporting Information). Flow cytometry data showed that CD4^+^/CD8^+^ T cells were significantly high in the MHC‐II vaccinated group (Figure 4e). Additionally, co‐administration of the MHC‐I and MHC‐II vaccines induced more proliferation of CD8^+^ short‐lived effector T cells (SLECs, CD127^‐^ KLRG1 ^+^ of CD8^+^ cells) than only the MHC‐I vaccine (Figure 4f), suggesting that MHC‐II antigen‐specific CD4^+^ T cells help proliferation and differentiation of short‐lived effector CD8^+^ T cells. The proliferation of regulatory T cells (T_reg_), which typically indicate the inhibition of immune responses, was significantly reduced in all vaccinated groups (Figure 4g). Antigen‐specific IFN‐γ and TNF‐α secretion from CD8^+^ T cells was significantly increased in the MHC‐I vaccinated group (Figure 4h, upper). On the other hand, immunization with the MHC‐II vaccine increased IFN‐γ and TNF‐α levels in CD4^+^ T cells (Figure 4h, lower). Interestingly, antigen‐specific CD8^+^ T cells secreted significantly higher levels of IFN‐γ and TNF‐α in the group receiving both MHC‐I and MHC‐II vaccines than MHC‐I‐only vaccination group in the tumor‐bearing mouse model, whereas there were no significant differences in the IFN‐γ and TNF‐α secretion by CD4⁺ T cells between the MHC‐II vaccination group and the MHC‐I/II co‐administration group. These results showed similar patterns to the results from healthy mice (Figure 3d–g), suggesting that co‐administration of these vaccines can induce an effective immune response and enhance anti‐cancer efficacy. To further validate neoantigen‐specific immune responses induced by mRNA‐based neoantigens in the tumor‐bearing mouse model, IFN‐γ ELISpot analysis was performed.

The results showed that the MHC‐I vaccine elicited MHC‐I antigen‐specific immune responses and the MHC‐II vaccine elicited MHC‐II antigen‐specific immune responses (Figure S7a−c, Supporting Information). Furthermore, the group that received co‐administration of the MHC‐I and MHC‐II vaccines showed significantly increased MHC‐I antigen‐specific immune responses than the group receiving only the MHC‐I vaccine, but not MHC‐II‐related immune responses. These results suggest the MHC‐II antigens not only stimulate CD4^+^ T cells to induce a type 1 helper T cell response but also activate CD8^+^ T cells to function as helper T cells.

### 2.5 mRNA Vaccine Reduces Tumor Recurrence After Surgery and Prophylactic Vaccination in a Mouse Colon Cancer Model

**Figure 5:**
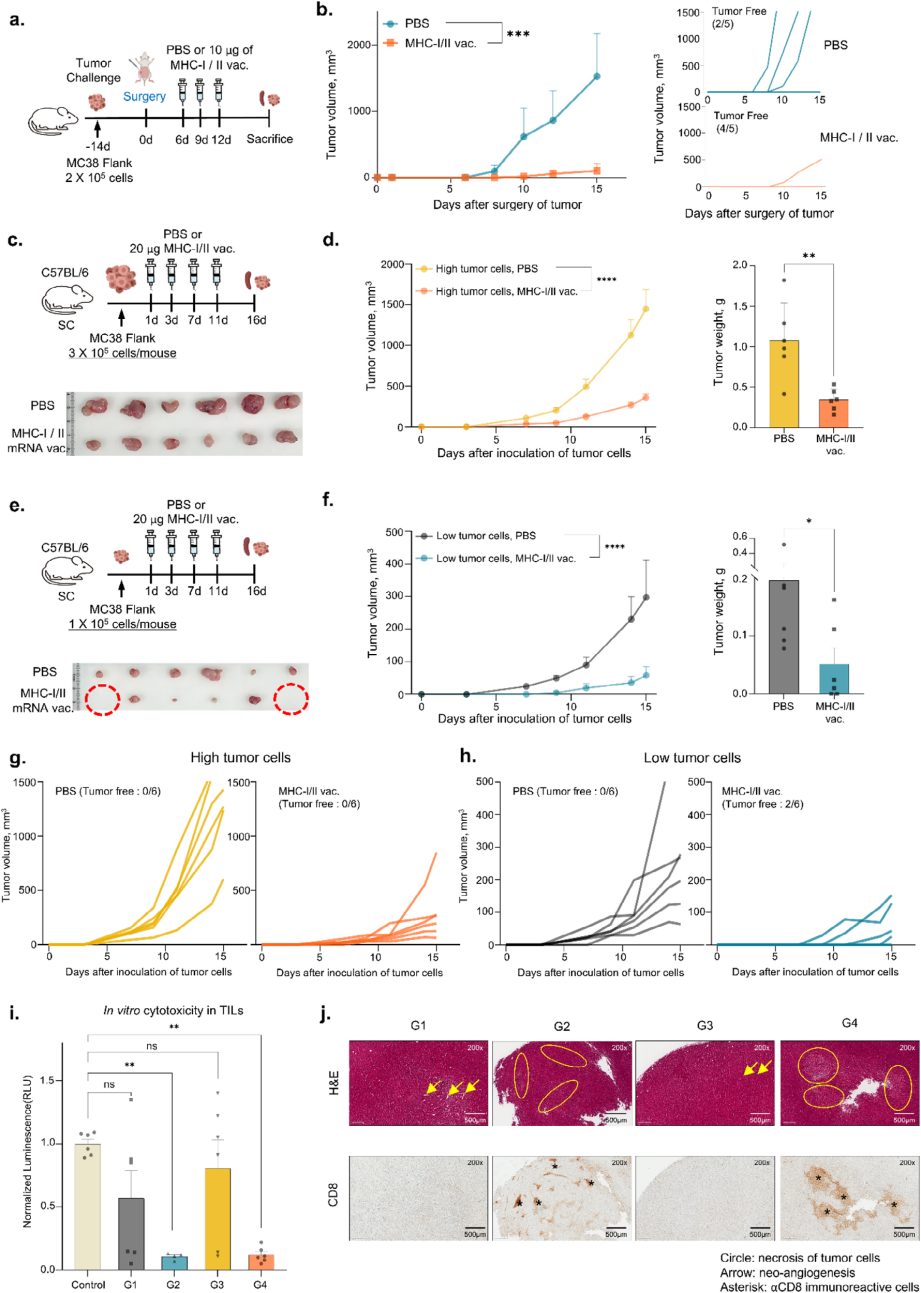
mRNA vaccine reduces tumor recurrence after surgery in a mouse colon cancer model and increase anti‐cancer efficacy in early colon cancer development. a) Experimental design of tumor recurrence inhibition study after surgical treatment. Mice were subcutaneously inoculated 2 × 10^5^ MC38 tumor cells. After 14 days, tumor tissues were surgically removed, and the mice were administered PBS or co‐administered MHC‐I and MHC‐II vaccines (n = 5 per group) on days 6, 9, and 12. b) Data of tumor growth monitoring until 15 days after surgical treatment and tumor images following PBS or mRNA vaccine treatment. c–h) Experimental design of anti‐cancer efficacy by mRNA vaccines transplanted with high number of tumor cells c,d, g) or low number of tumor cells e,f, h). C57BL/6J mice were subcutaneously injected with MC38 colon cancer cells (1 × 10^5^ [c] or 3 × 10^5^ cells per  mouse [e]). Tumor‐bearing mice were subcutaneously immunized with PBS or 20 µg of MHC‐I/II mRNA vaccine on days 1, 3, 7, and 11 (n = 6 per group). Tumor weight and growth monitoring until 16 days after tumor inoculation in high tumor cells d) and in low tumor cells f). Individual tumor growth curve in high tumor cells g) and in low tumor cells h). i) In vitro CTL assay of TILs isolated from mice immunized with MHC‐I/II vaccine. TILs isolated from mice were mixed with 5 × 10^4^ MC38‐Luc tumor cells (Effector cell: Target cell = 5:1). After 16 h, luciferase activity was measured for tumor cell viability. Relative normalized luminescence (RLU) was calculated as the luminescence of each group compared with the control group (MC38‐Luc cells without effector cells). G1, The group of treatment with PBS in low tumor cells inoculated mice; G2, The group of treatment with mRNA vaccine in low tumor cells inoculated mice; G3, The group of treatment with PBS in high tumor cells inoculated mice; G4, The group of treatment with mRNA vaccine in high tumor cells inoculated mice. h) Histological features in hematoxylin and eosin (H&E) stained MC38 tumor sections (upper panel) and immunohistochemical staining for CD8 (lower panel) in the tumor tissues. Data of tumor growth curve shown as the mean ± standard error of the mean (SEM), analyzed using two‐way ANOVA with Dunnett's multiple comparisons test. In vitro cytotoxic assay results (mean ± SEM) are calculated using unpaired two‐tailed Student's *t*‐test (****p < 0.0001, ***p < 0.001, **p < 0.01, *p < 0.05).

To test the effects of the mRNA vaccine on preventing tumor recurrence, tumor‐bearing mice were treated using tumor resection surgery and injected with PBS or co‐administered the MHC‐I and MHC‐II mRNA vaccines (**Figure** **5**a). Compared to the PBS‐treated group, the mRNA vaccine group exhibited a significant reduction in tumor recurrence (Figure 5b). The antigen‐specific IFN‐γ was secreted by spleen cells isolated from vaccinated mice (Figure S8, Supporting Information). These results show that mRNA vaccines encoding neoantigens can reduce the recurrence burden of colon cancer by antigen‐specific T cell responses. Additionally, the anti‐cancer efficacy of prophylactic vaccination was tested by administering PBS or MHC‐I/MHC‐II vaccines once per week for 3 weeks before tumor inoculation (Figure S9a, Supporting Information).

Groups receiving the mRNA‐based neoantigen vaccine exhibited significantly reduced tumor growth and a higher number of tumor‐free mice compared to the control group (Figure S9b, Supporting Information). Additionally, IFN‐γ ELISpot analysis confirmed the induction of neoantigen‐specific IFN‐γ secretion, indicating a robust immune response following prophylactic vaccination (Figure S9c, Supporting Information).

### 2.6 Anti‐Cancer Efficacy of mRNA Vaccines in Early Colon Cancer Development

The efficacy of anti‐cancer vaccines varies based on the stage of cancer development.^[^ ^16^ ^]^ C57BL/6 mice were inoculated with MC38 cells ranging from 1 × 10^5^ cells to 3 × 10^5^ cells to investigate the efficacy of the mRNA vaccines at different stages of cancer development (Figure 5c–f). Tumor volume and weight were significantly inhibited in the vaccine‐treated group compared with the PBS‐treated group, and the anti‐tumor effect was greater in the group transplanted with a low low‐dose cancer cells (Figure 5c–h; Figure S10a, Supporting Information). In particular, two of six mice in the mRNA vaccine group were tumor‐free (Figure 5e,h), indicating that anti‐cancer vaccine administration was more effective in earlier cancer stages.

A cytotoxicity assay was performed to evaluate the cytotoxic function of lymphocytes. TILs isolated from mice administered with PBS or the mRNA vaccine were mixed with firefly luciferase‐transduced MC38 colon cancer cells (MC38‐luc). The viability of MC38‐luc cancer cells was significantly decreased in the mRNA vaccine‐treated group (Figure 5i). These results show that the mRNA vaccine can activate the cytotoxic function of lymphocytes. Furthermore, Splenocytes and TILs from all mice administered with the mRNA vaccine exhibited antigen‐specific T cell responses (Figure S10b,c, Supporting Information). As tumor‐infiltrating CD8^+^ T cells are a crucial factor in cancer immunotherapy, TILs were isolated from vaccinated mice to analyze the tumor‐infiltrating T cell population. The results of the flow cytometry analysis showed significantly more CD8^+^ T cells in the mRNA vaccination group than in the PBS group (Figure S11a, Supporting Information). Additionally, the mRNA vaccine significantly reduced the proliferation of suppressor T cells (CD25^high^CD4^+^) within tumor tissues (Figure S11b, Supporting Information).

Tumor tissues were subjected to hematoxylin and eosin (H&E) staining, and immunohistochemical analysis was performed to assess histological differences in tumors between the PBS‐treated and mRNA‐treated groups. Consequently, the necrotic areas in the H&E staining were equally immunostained using a mouse anti‐CD8 antibody in the immunohistochemistry (IHC) staining, suggesting that CD8^+^ T cells infiltrated the tumor and influenced the death of tumor cells (Figure 5j).

### 2.7 Anti‐Cancer Efficacy Based on the Timing of mRNA Vaccine Treatment

**Figure 6:**
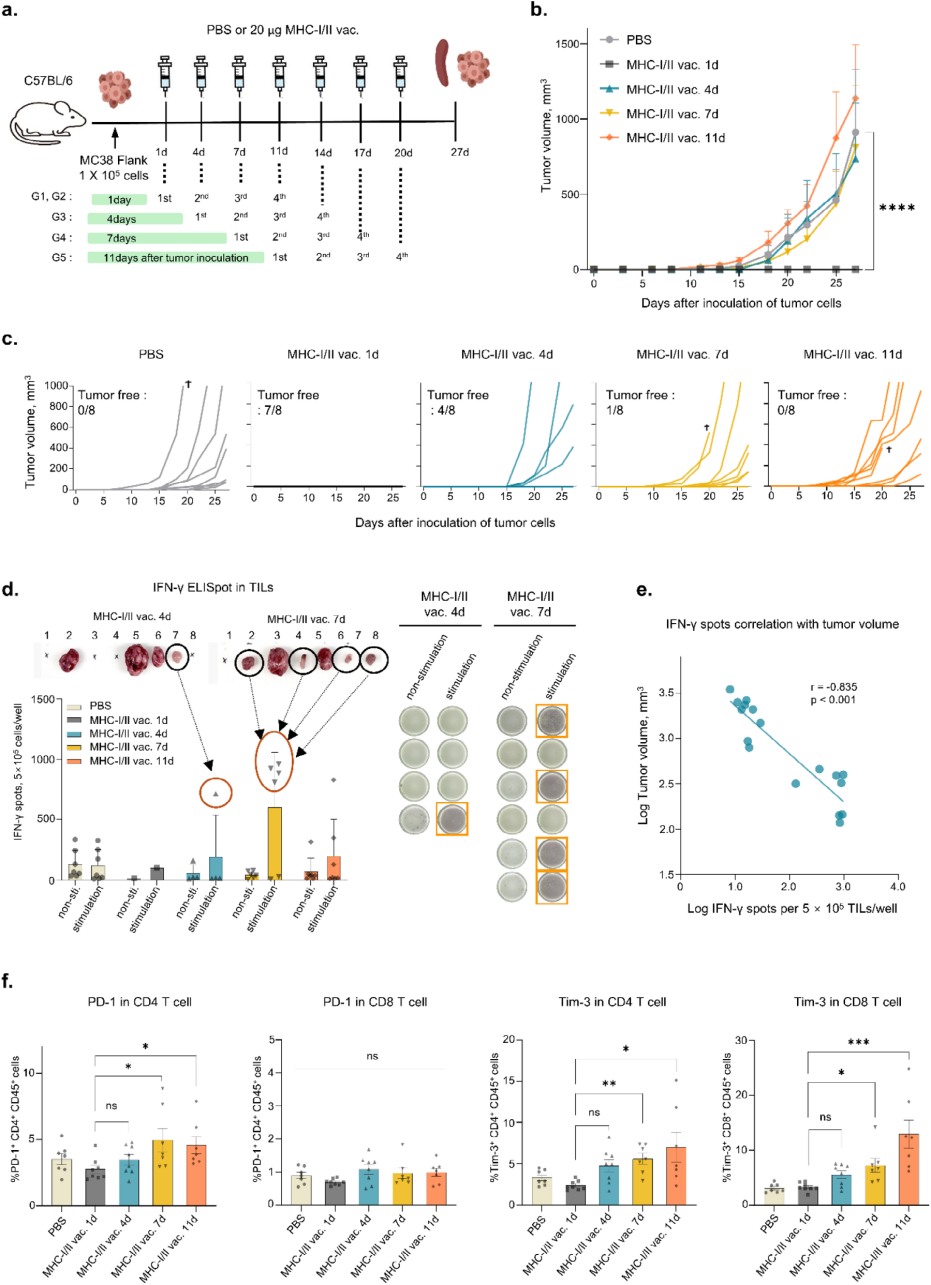
Early‐stage vaccination produces a robust anti‐cancer effect. a) Experimental design of in vivo colon cancer model. MC38 cells (1 × 10^5^ cells) were injected into C57BL/6J mice. Mice in each group were primarily immunized with 20 µg of MHC‐I/II mRNA vaccine on days 1, 4, 7, and 11, followed by four doses every 3–4 days. b) Growth kinetics of MC38 tumor immunized with MHC‐I/II vaccine depending on vaccination time (n = 8). c) Individual tumor growth data. d) IFN‐γ ELISpot results for investigation of tumor infiltration of neoantigen‐specific T cells. After TILs isolated from mice were re‐stimulated with pooled MHC‐I/II peptide (CNM‐I‐08, CNM‐I‐13, CNM‐I‐19, CNM‐II‐01, and CNM‐II‐04), antigen‐specific IFN‐γ spots were compared to tumor size. e) Correlation analysis of neoantigen specific‐secretion of IFN‐γ in TILs with tumor volume. The *x*‐axis and *y*‐axis were log‐transformed for analysis, and correlations were analyzed using Pearson's analysis (*r* = −0.835, *p* < 0.001). *p* < 0.05 is regarded as statistically significant. f. Flow cytometric analysis of immune checkpoint (PD‐1 and Tim‐3) in CD4^+^ and CD8^+^ T cells in spleen cells. Data of flow cytometry are shown as the mean ± standard error of the mean (SEM), analyzed using one‐way ANOVA with Dunnett's multiple comparisons test and unpaired two‐tailed Student's *t*‐test (****p < 0.0001, ***p < 0.001, **p < 0.01, *p < 0.05).

A previous study has shown that anti‐cancer efficacy may vary depending on the timing of cancer vaccine treatment.^[^ ^17^ ^]^ The primary mRNA vaccination was administered to MC38‐bearing mice at different time points (1, 4, 7, and 11 days after inoculation of tumor cells) to confirm the efficacy of the cancer vaccine based on the vaccination time (**Figure** **6**a). Compared with other treatment groups, the earliest mRNA vaccine group exhibited significant tumor growth inhibition and tumor disappearance in seven out of eight animals (Figure 6b,c).

IFN‐γ ELISpot analysis was performed using TILs to confirm the induction of antigen‐specific T cell responses based on vaccination timing. Mice in the late vaccination group, where antigen‐specific immune responses were induced in TILs, exhibited smaller tumor sizes (Figure 6d). Furthermore, the correlation between antigen‐specific T cell‐related IFN‐γ secretion and anti‐tumor activity was assessed using Pearson's correlation coefficient, which showed a negative correlation (r = −0.835, *p* < 0.001) between antigen‐specific IFN‐γ secretion and tumor growth (Figure 6e). These findings indicate that the infiltration of neoantigen‐specific T cells into tumor tissues is a critical factor for improving anti‐cancer efficacy.

Despite the excellent immune activation achieved through cancer vaccine treatment, T cell exhaustion remains an obstacle to successful anti‐cancer immunotherapy.^[^ ^18^ ^]^ The results of flow cytometric analysis for T cell exhaustion markers showed that the early immunized group exhibited lower expression of PD‐1 and Tim‐3 in CD4^+^or CD8^+^ T cells (Figure 6f). The expression of Tim‐3 in CD4^+^ or CD8^+^ T cells appeared to gradually increase with delay in the vaccination period. These results suggested that early vaccination with mRNA cancer vaccines and combination therapy with ICIs are necessary for efficient anti‐cancer treatment.

### 2.8 mRNA Vaccination Induced Persistent MHC‐I and MHC‐II Antigen‐Specific Immune Responses and Reduced Tumor Recurrence Burden

**Figure 7:**
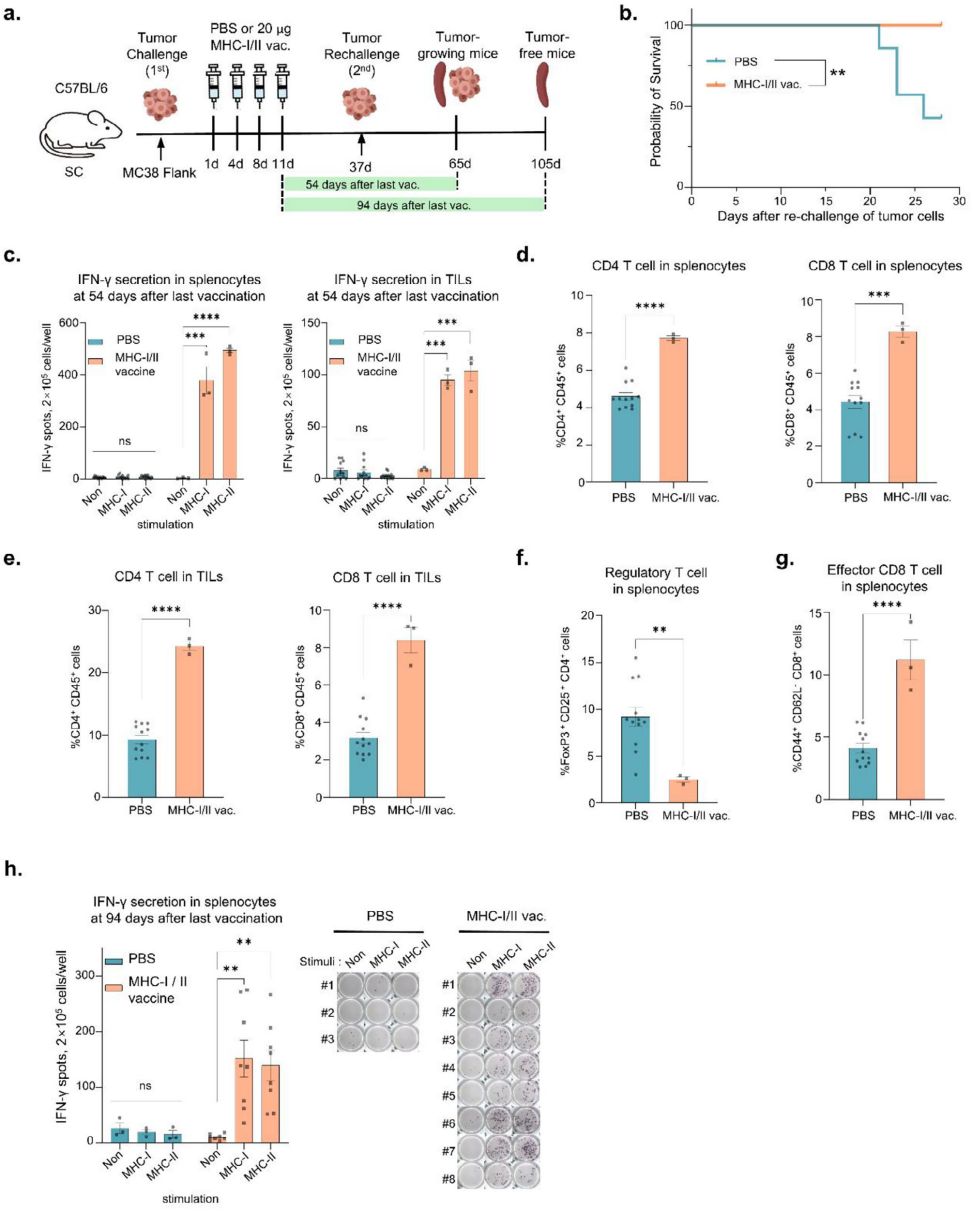
mRNA vaccines prevent tumor recurrence and induced persistent MHC‐I and MHC‐II antigen‐specific immune responses. a) Experimental design of in vivo tumor re‐challenge. After MC38‐bearing mice were administered PBS or MHC‐I/II vaccines, tumor‐free mice in each group were re‐challenged with MC38 tumor cells on 37 days after 1st challenge of tumor cells (n = 7, PBS‐treated group; n = 9, mRNA‐vaccinated group). Tumor‐growing mice (n = 4; PBS‐treated group, n = 1; mRNA‐vaccinated group) were euthanized at 28 days after tumor re‐challenge. b) Survival rates were monitored until 28 days after tumor re‐challenge. Statistics of survival curve using log‐rank Mantel‐Cox test (**p < 0.01). c) IFN‐γ ELISpot analysis of neoantigen‐specific T cell response in the spleen (left) and TILs (right) at 54 days after last vaccination. d–g) Flow cytometric analysis for populations of CD4^+^ T cells (CD4^+^CD45^+^) and CD8^+^ T cells (CD8^+^CD45^+^) in spleen cells d) and TILs (e), regulatory T cells (f, FoxP3^+^CD25^+^), and effector CD8^+^ T cells (g, CD44^+^CD62L^‐^) in spleen cells. h. IFN‐γ ELISpot analysis of neoantigen‐specific T cell response in the spleen at 94 days after last vaccination. The right panel shows the image of ELISpot analysis. IFN‐γ ELISpot and flow cytometric analysis were performed in triplicate per mouse. Data shown as mean ± SEM, analyzed using one‐way ANOVA with Dunnett's multiple comparisons test and using unpaired two‐tailed Student's *t*‐test (****p < 0.0001, ***p < 0.001, **p < 0.01, *p < 0.05).

Cancer vaccines can prevent tumor recurrence or metastasis by inducing the differentiation of memory T cells.^[^ ^19^ ^]^ To determine whether the mRNA vaccine confers protection by inducing persistent immune memory against cancer antigens, tumor‐free mice were re‐challenged with colon cancer cells following the initial mRNA vaccination (**Figure** **7**a). In the mRNA vaccine‐treated group, all mice survived up to 30 days post‐rechallenge, whereas the PBS‐administered control group exhibited significantly lower survival rates (Figure 7b). Isolated splenocytes and TILs were subjected to ELISpot analysis for IFN‐γ secretion 54 days after the last vaccination to confirm the persistence of antigen‐specific immune responses induced by mRNA vaccines (Figure 7c). Additionally, mRNA vaccine administration increased the population of CD4^+^ or CD8^+^ T cells in both the spleen and TILs (Figure 7d and 7e). In the spleen of mice treated with the mRNA vaccine, a decrease in T_reg_ cells (FoxP3^+^CD25^+^CD4^+^ cells) and a concomitant increase in effector memory CD8^+^ T cells (CD44^+^CD62L^‐^ CD8^+^ cells) were confirmed at 54 days after last vaccination (Figure 7f,g), indicating that the mRNA vaccines maintained long‐term immune responses by generating memory T cells. Furthermore, the neoantigen‐specific T cell responses in splenocytes were still present 94 days after the last vaccination (Figure 7 h). Consequently, mRNA vaccination induced persistent MHC‐I and MHC‐II antigen‐specific immune responses in splenocytes and TILs, respectively.

### 2.9 Synergistic Effect of Anti‐Cancer Efficacy by Combination Treatment with mRNA Vaccine and ICIs

**Figure 8:**
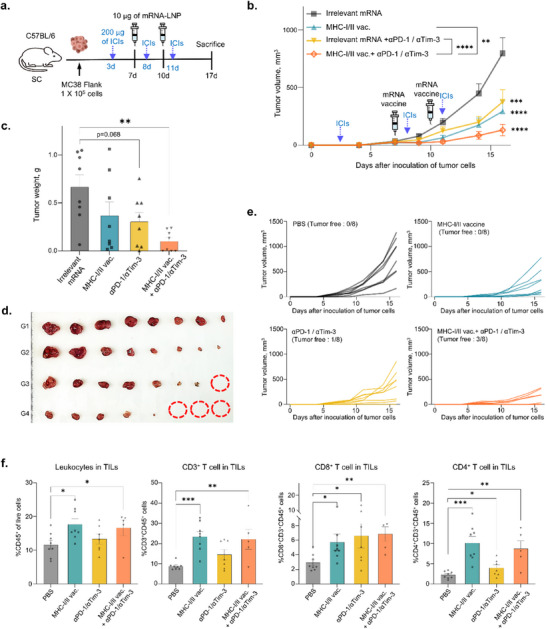
Combination therapy of mRNA vaccines with immune checkpoint inhibitors synergistically inhibits tumor growth. a) Combination treatment involving the MHC‐I/II vaccine with immune checkpoint inhibitors, anti‐PD‐1, and anti‐Tim‐3. MC38 tumor‐bearing mice (n = 8 per group) were injected with irrelevant mRNA (mRNA encoding GFP), MHC‐I/II vaccine, anti‐mouse PD‐1/Tim‐3 antibodies, and combination therapy. b) Tumor growth was monitored until 17 days after tumor cell inoculation (mean ± SEM; ****p < 0.0001, **p < 0.01, Two‐way ANOVA with Dunnett's multiple comparisons test). c) Tumor weights were measured from each mouse at 17 days after inoculation. d) Tumor image. e) Individual data of tumor growth curve. f) Flow cytometric analysis for populations of leukocytes (CD45^+^ cells), CD3^+^ T cells (CD3^+^CD45^+^ cells), CD4^+^ T cells (CD4^+^CD3^+^CD45^+^), and CD8^+^ T cells (CD8^+^ CD3^+^CD45^+^) in TILs. Data is shown as the mean ± SEM, analyzed using one‐way ANOVA with Dunnett's multiple comparisons test and using unpaired two‐tailed Student's *t*‐test (****p < 0.0001, ***p < 0.001, **p < 0.01, *p < 0.05).

Given that mRNA vaccines increased the expression of immune checkpoint molecules, a combination treatment of ICIs and cancer vaccines was performed in a colon cancer model. C57BL/6 mice were transplanted with 1 × 10^5^ cells of MC38 colon cancer cells in the right flank, then administered with 200 µg of ICIs (anti‐mouse PD‐1 and Tim‐3 antibody) on 3, 8, and 11 days or with 10 µg of mRNA vaccine on 7 and 10 days, or both (**Figure** **8**a). Notably, tumor growth was significantly reduced in the mRNA vaccine and ICIs‐treated groups compared to the control group (Irrelevant mRNA). The combined treatment group exhibited a more pronounced reduction in tumor growth than either the mRNA vaccine or ICIs alone (Figure 8b,c). Moreover, in the combination therapy group, tumors completely regressed in three out of eight mice, highlighting a synergistic effect on tumor growth inhibition (Figure 8d,e). To further investigate the impact of combination therapy on immune cell infiltration, flow cytometric analysis of tumor‐infiltrating lymphocytes (TILs) was conducted. The results revealed that treatment with both the mRNA vaccine and ICIs significantly increased the presence of immune cells, including CD45^+^, CD3^+^, CD8^+^, and CD4^+^ T cells, within the tumor microenvironment (Figure 8f). This suggests that the combination therapy promotes immune cell infiltration into the tumor, thereby enhancing anti‐tumor immunity. Furthermore, to assess the safety of the mRNA‐based cancer vaccine, histopathological analysis was performed on major organs, including the heart, kidney, liver, lung, and spleen (Figure S12a, Supporting Information). All groups, including PBS, MHC‐I, MHC‐II, and MHC‐I/II vaccine groups, showed no significant changes or pathological findings. Specifically, no evidence of pericarditis, calcification, muscular degeneration, immune cell infiltration, or abnormal heart diameter was observed in the heart. Similarly, the kidney, liver, lung, and spleen showed no signs of inflammation, tissue structural changes, fibrosis, or other pathological alterations (Figure S12b, Supporting Information). These findings indicate that the mRNA‐based cancer vaccine is safe and does not cause detectable tissue damage or toxicity in evaluated organs.

## 3 Discussion

Clinical trials have revealed promising results regarding the efficacy of PCVs in treating cancer.^[^ ^20^ ^]^ In November 2023, the Korean Ministry of Food and Drug Safety published the guideline “Considerations on the Development of Personalized Neoantigen‐Coded Therapy Products” announcing that efficacy testing “can be evaluated by selecting mouse tumor cell lines similar to the cancer type to be treated, screening for neoantigens using the same strategy as in humans, and making preparations using the same manufacturing process.”^[^ ^21^ ^]^ Based on this guideline, the study investigated the optimal conditions for PCVs in treating tumors using cell lines of a mouse with colon cancer to predict neoantigens and performing experiments under various conditions that were impossible in human clinical trials.

In this study, neoantigens in a mouse model of colon cancer were identified using a proprietary antigen prediction technique, and MHC‐I and MHC‐II neoantigens, which induced antigen‐specific T cell responses, were selected. This result indicates that the mRNA platform, including multi‐neoantigens, should be used for PCV production rather than peptide vaccines, given that the predictive rate of neoantigens is only 6% in most studies.^[^ ^22^ ^]^ The key advantage of mRNA‐based vaccines lies in their ability to encode multiple neoantigens within a single construct, significantly increasing the likelihood of generating a robust and effective immune response. This multi‐epitope approach is particularly advantageous in PCVs, as it allows rapid and tailored production based on each patient's unique tumor mutation profile, enhancing the potential for personalized treatment.^[^ ^23^ ^]^

For mRNA‐based MHC‐I neoantigens, longer sequences of 27 aa induced immune responses stronger than shorter neoantigenic sequences of 8–10 aa, suggesting that platform design based on antigen length can also influence immune effectiveness.^[^ ^13^, ^24^ ^]^ Moreover, mRNA not only delivers antigens but also stimulates innate immune responses, thereby boosting adaptive immunity.^[^ ^25^ ^]^ This dual function of mRNA, as both an antigen source and an immune stimulant, aligns with our findings, where the mRNA platform demonstrated superior immune activation. Notably, we observed that certain neoantigens, such as CNM‐I‐13 and CNM‐I‐19, showed strong immunogenicity in peptide‐based immunization but markedly reduced responses when delivered as mRNA‐encoded short 10‐mer sequences. This discrepancy is likely due to inefficient epitope processing by the proteasome, which does not always generate the exact minimal epitope required for MHC‐I presentation. In contrast, extended sequences such as 27‐mers facilitate more efficient intracellular processing, thereby improving antigen presentation and activation of CD8 positive T cells. Our observations are supported by previous reports,^[^ ^10^, ^13^ ^]^ which demonstrated that mRNA vaccines encoding long peptides with centrally located epitopes elicited more robust immune responses and effective tumor control in clinical and preclinical studies. These findings highlight the importance of rational vaccine design incorporating flanking regions around the predicted epitope to ensure effective antigen processing and presentation.

Co‐administration of MHC‐I‐ and MHC‐II‐restricted neoantigens may induce better antigen‐specific CD8 T cell related immune responses and anti‐tumor efficacy than only MHC‐I‐restricted neoantigens. This phenomenon likely stems from the multifunctional immune response of CD4^+^ T cells activated through the MHC‐II pathway.^[^ ^13^, ^26^ ^]^ CD4^+^ T cells facilitate the activation of CD8^+^ T cells and secrete cytokines that recruit other immune cells, such as macrophages and dendritic cells, further enhancing the anti‐tumor activity. Recent evidence of cytotoxic CD4^+^ T cells highlights their direct tumor‐killing potential, expanding their functional importance.^[^ ^27^ ^]^ Additionally, MHC‐II expression on tumor cells has been associated with improved T cell infiltration and enhanced immunotherapy outcomes.^[^ ^28^ ^]^ These findings underscore the value of MHC‐II‐restricted neoantigens in cancer vaccines, as they can simultaneously activate CD4^+^ T cells and reshape the tumor microenvironment (TME), thereby enhancing the overall anti‐tumor immune response. Moreover, a balanced increase in CD8^+^ T cell and CD4^+^ T cell‐mediated immune responses could be achieved to obtain effective anti‐cancer effects by the combined treatment of MHC‐I and MHC‐II neoantigens. The key to immunity is “balance,”^[^ ^29^ ^]^ and this principle is also important in cancer treatment through cancer vaccines to maintain the homeostasis of the body. Ensuring the balanced activation of CD8^+^ and CD4^+^ T cells is essential for achieving successful tumor eradication. Additionally, selecting immunogenic neoantigens is important for eliciting strong anti‐cancer effects. The MHC‐I and MHC‐II neoantigens predicted in this study effectively activated CD8^+^ and CD4^+^ T cells, respectively, indicating that the neoantigen prediction algorithm used in this study is not inferior to traditional prediction methods.

Another important aspect of these findings is that the vaccination timing can significantly impact the anti‐tumor effect of a PCV. The PCV developed in this study had a higher anti‐tumor effect when vaccinated with fewer cancer cells and at an earlier cancer stage. In clinical practice, different patients with cancer have different cancer sizes and progression, and the induction of immune responses and anti‐tumor effects of various cancer antigens may also be different.^[^ ^30^ ^]^ Thus, rapid administration of PCVs will be linked to their anti‐tumor efficacy, and providing vaccines that can be quickly manufactured for patients with early‐stage cancer is important. Moreover, this study showed that PCVs, in combination with ICIs, are sufficient anti‐tumor agents, even when administered later after cancer cell challenge in mice. This finding strongly highlights the need for combination therapy involving PCVs and other anti‐tumor drugs.^[^ ^31^ ^]^ As shown by Rojas et al.,^[^ ^12a^ ^]^ various combinations of surgical cancer removal, ICI treatment, and chemotherapy—rather than relying on PCV monotherapy—help improve the survival rate of patients with cancer.

Many conventional anti‐tumor drugs are not completely curative, as cancers become resistant, relapse, or metastasize.^[^ ^32^ ^]^ However, the study showed that mice immunized with the mRNA‐based PCV after surgical tumor removal had a significantly lower recurrence rate than unimmunized mice, consistent with results showing superior recurrence‐free survival among responders immunized with PCV.^[^ ^10^, ^12^ ^]^ These findings strongly suggest that PCVs may eventually address the fear of recurrence and metastasis in patients with cancer. Nevertheless, this research has some limitations, and collaboration across multiple disciplines is critical, particularly in developing rapid and accurate cancer biomarkers and advancing in bioinformatics to improve the low neoantigen prediction rate.

In conclusion, this study demonstrates that mRNA‐based PCVs hold great promise in reducing the risk of cancer recurrence and metastasis by inducing the generation of memory T cells. The enhanced anti‐tumor efficacy of PCVs when combined with ICIs was also confirmed in tumor‐bearing mouse models, suggesting that various combination treatments can further improve the therapeutic potential of PCVs. The findings emphasize the importance of administering PCVs as early as possible after cancer onset to maximize their effectiveness. Considering these unique characteristics of PCVs, prioritizing PCV use in patients with early‐stage cancer, such as those with stage 1 or 2, and minimizing the time from neoantigen identification to vaccine administration is crucial. To realize the full potential of PCVs and bring hope to patients with cancer seeking a cure, concerted efforts are needed to expedite neoantigen prediction, innovate manufacturing processes, and streamline regulatory pathways to accelerate product delivery.

By addressing these key challenges, paving the way for the widespread implementation of PCVs as a powerful tool in the fight against cancer is possible, offering renewed hope for patients and their families.

## 4 Experimental Section

### Peptides and Antibodies

Each neoantigen candidate was chemically synthesized as a peptide (Peptron, Daejeon, Korea). The peptides were synthesized in lengths of 8–10 mers or 27 mers. SM‐102, distearoylphosphatidylcholine (DSPC), polyethylene glycol (PEG), and cholesterol for LNP formulation with mRNA were purchased from Avanti Polar Lipid, Inc. (Birmingham, AL, USA). Mouse anti‐CD4 APC‐Cy7, fluorescein isothiocyanate antibodies (Cat.100414, 130 308), anti‐CD8‐BV421 antibody (Cat.100738), anti‐CD45‐BV650 antibody (Cat.103151), anti‐TNFa‐fluorescein isothiocyanate antibody (Cat.506304), anti‐CD127‐APC antibody (Cat.135012), anti‐KLRG1‐BV605 antibody (Cat.138419), anti‐CD62L‐PE Cy7 antibody (Cat.104418), anti‐PD‐1‐PE antibody (Cat.135206), anti‐PD‐1‐APC‐Cy7 antibody (Cat.135224), anti‐CTLA‐4‐PE antibody (Cat.106306), and anti‐Tim‐3‐BV421 antibody (Cat.119723) were purchased from Biolegend (San Diego, CA, USA). Mouse anti‐CD8‐Percp‐Cy5.5 (Cat.45‐0081‐82), anti‐CD8‐PE‐Cy7 antibody (Cat.25‐0081‐82), anti‐CD45‐Percp‐Cy5.5 antibody (Cat.45‐0451‐81), anti‐IFN‐γ‐PE antibody (Cat.12‐7311‐82), and anti‐IFN‐γ‐APC antibody (Cat.17‐7311‐82) were purchased from Invitrogen (Waltham, MA, USA). Mouse anti‐FoxP3‐PE antibodies (Cat.560408) were purchased from BD Biosciences (San Diego, CA, USA). The epitope (SYALILRTIL, CNM‐13‐06)‐specific tetramer was produced as described previously.^[^ ^33^ ^]^ In brief, recombinant H2‐D^b^ protein obtained from *Escherichia coli* BL21 (DE3) was refolded in the presence of the SYALILRTIL peptide and human β2m, biotinylated with biotin‐protein ligase BirA, and then purified with Superdex‐75 gel filtration column (GE Healthcare Life science). Purified monomer was tetramerized with R‐phycoerythrin conjugate (SAPE; Thermo Fisher Scientific, USA).

### Cell Cultures and Mice

MC38 murine colon adenocarcinoma cells were purchased from Sigma‐Aldrich (St. Louis, MO, USA). MC38 cells were cultured in Dulbecco's modified Eagle's medium supplemented with 10% fetal bovine serum (Gibco, Grand Island, NY, USA), penicillin (100 IU mL^−1^), streptomycin (100 µg mL^−1^), and amphotericin B (0.25 µg mL^−1^) and grown in a humidified incubator with 5% carbon dioxide (CO_2_) at 37 °C. All animal experimental procedures were approved by the Institutional Animal Care and Use Committee of the Catholic University of Korea (CUK‐IACUC‐2023‐034). Female C57BL/6J mice (6–8 weeks old) were purchased from DBL Co., Ltd. (Chungcheongbuk‐do, South Korea). They were housed in a temperature‐controlled room and maintained on a 12‐h light/dark cycle with free access to food and sterile water under specific pathogen‐free conditions.

### Exome and Transcriptome Sequencing and Tumor‐Specific Antigen (TSA) Prediction

Sequencing on normal colon tissue and cancer cells was performed to identify TSAs unique to cancer tissues through comparative analysis with normal tissues and design effective epitopes. Exome and mRNA‐seq libraries were constructed using Agilent's SureSelectXT Library Prep Kit and the TruSeq Stranded mRNA Library Prep Kit, respectively. Two biological replicates were prepared for mRNA‐seq. Sequencing was conducted on the Illumina Novaseq 6000 platform to generate 101‐bp paired‐end reads following the manufacturer's protocol. The read data were processed with Trimmomatic to remove low‐quality bases and sequencing artifacts using the parameters TruSeq3‐PE‐2.fa:2:30:10:2 HEADCROP:5 TRAILING:20 MINLEN:75. TSAs were identified as inducers of specific immune responses in tumor tissues based on SNVs and small InDels from the Exome data and gene fusion information from the RNA‐seq data. Figure 1b illustrates the overall process of identifying cancer‐specific variants. The reference genome GRCm39 from the Ensembl database was used for TSA discovery, annotation, and expression profiling.

For TSA identification using Exome data, the trimmed and filtered reads were mapped to GRCm39 using BWA‐MEM2,^[^ ^34^ ^]^ and PCR duplicates were removed using the MarkDuplicate module in Picard tools (http://broadinstitute.github.io/picard/). As the tumors were generated using a distinct cancer cell line and not typical somatic mutations, germline variant callers GATK HaplotypeCaller^[^ ^35^ ^]^ and Samtools^[^ ^36^ ^]^ were employed for variant calling. The identified variants were filtered using VCFtools^[^ ^37^ ^]^ to exclude those with variant quality < Q30, missing data, multi‐allelic loci, and read depth < 30. Gene‐related information for the variants was obtained using snpEff^[^ ^38^ ^]^ and Variant Effect Predictor^[^ ^39^ ^]^ with Ensembl 108 annotation. From the annotation data, non‐synonymous mutations present exclusively in tumor tissues and absent in normal tissues, identified by both variant callers, were selected. These variants were considered as candidate TSAs for epitope design.

For gene fusion identification using RNA‐seq, reads from the two biological replicates were mapped with STAR aligner^[^ ^40^ ^]^ and identified gene fusions using ARRIBA^[^ ^41^ ^]^ and STAR‐Fusion.^[^ ^42^ ^]^ Gene fusions were consistently identified by both replicates, and both tools were selected. These gene fusions were matched to the assembly results obtained using Trinity^[^ ^43^ ^]^ to confirm their presence in hisat2.^[^ ^44^ ^]^ Confirmed gene fusions, SNVs, and InDels identified from exome sequencing were used as candidate TSAs for epitope design.

Antigen prioritization was conducted to design epitopes capable of inducing specific immune responses against tumor tissues from the identified candidate TSAs. Initially, featureCounts from the subread package^[^ ^45^ ^]^ on the mRNA‐seq alignment results obtained with the STAR aligner were used to select TSAs with sufficient expression levels. Expression levels in counts per million were normalized and ranked using edgeR,^[^ ^46^ ^]^ after which candidate TSAs were associated with genes in the bottom 10% of expression levels. Furthermore, to exclude candidate TSAs whose aa sequences are similar to those of self‐genes, potentially failing to induce an immune response, TSAs indistinguishable were filtered out from self‐gene aa sequences based on Ensembl 108 annotation. For the remaining candidate TSAs, netMHCpan^[^ ^47^ ^]^ and netMHC‐IIpan^[^ ^47^ ^]^ were used to assess binding affinity to the MHC alleles H‐2‐Dd, H‐2‐Kd, H‐2‐Kb, and H‐2‐Db in the C57BL/6 mouse strain for both MHC‐I and MHC‐II. Strong binders with a binding strength rank within the top 1% were filtered out. Using NetChop,^[^ ^48^ ^]^ the number of cleavage points within the designed epitopes was also examined. Considering the individual characteristics and sources (SNV, InDel, and gene fusion) of the candidate epitopes, the final candidate epitopes for MHC‐I and MHC‐II, which were expected to potentially induce specific immune responses against the tumor, were manually selected. In contrast to MHC‐I epitope design, the approach for designing MHC‐II epitopes entails systematically extracting the 13‐mer flanking sequences on either side of the target coordinate, which is then utilized as the epitope.

### mRNA Construct and Synthesis

The neoantigen sequences were inserted into the pCUK3‐1 mRNA platform backbone, as described in a previous study.^[^ ^49^ ^]^ Additionally, the neoantigen sequences were located between a signal peptide and an MITD sequence to facilitate efficient antigen presentation, as in previous studies.^[^ ^11^, ^15^ ^]^ The epitopes were designed in the form of pentamer or hexamer for each mRNA construct, and each epitope was designed by connecting with a glycine‐serine aa linker. The plasmid deoxyribonucleic acid was purified using an endotoxin‐free purification kit (Nucleo bond Xtra Midi Plus EF kit, MACHEREY‐NAGEL, Düren, Nordrhein‐Westfalen, Germany). It was prepared using a linearization process using Not I endonuclease (Enzynomics, Daejeon, South Korea). The mRNA was produced using an EZ T7 High‐Yield in vitro Transcription kit (Enzynomics) and a Cap 1 capping analog (SMARTCAP, ST PHARM, Gyeonggi‐do, South Korea). Wild‐type uridine was replaced with N1‐methyl‐pseudouridine‐5′‐triphosphate (m1ΨTP; TriLink, USA). The IVT mRNA mixture was purified via LiCl precipitation after DNase I (Enzynomics) treatment. dsRNA in the mRNA mixture was removed using the cellulose resin.^[^ ^50^ ^]^

### mRNA‐LNP Formulation

All lipid components (molar ratio of 50:10:38.5:1.5, Ionizable cationic lipid:DSPC:Cholesterol: DSPE‐PEG) were dissolved in chloroform‐methanol solution (1:1, v/v) and then dried under pressure to form a thin film for long‐term storage. The lipid mixture and mRNA were dissolved in absolute ethanol and citrate buffer (pH 4.0), respectively.^[^ ^51^ ^]^ Subsequently, the lipid mixture and mRNA were formulated at a ratio of 1:3 (v/v) using a microfluidic channel (Encell master, Enparticle, Busan, South Korea). The mRNA‐LNP solution was serially centrifuged for buffer exchange via ultrafiltration (30 kDa, Merck Millipore, Burlington, MA, USA).

### mRNA/mRNA‐LNP Characterization

RNA quality was confirmed via agarose gel electrophoresis. mRNA was quantified using absorbance at 260 nm, and the purity was confirmed using the Abs.260/Abs.280 ratio. Product‐related dsRNA was measured using an anti‐dsRNA‐based dot blot assay.^[^ ^50^ ^]^ The sizes and distributions of mRNA‐LNPs were measured using dynamic light scattering (Zetasizer 2000, Malvern Panalytical Ltd., Malvern, UK). The encapsulation efficiency was analyzed using the agarose gel electrophoresis method and Ribogreen assay (Quanti‐iT RiboGreen RNA assay kit, Invitrogen, Waltham, MA, USA), following the manufacturer's instructions.

### In Vivo Immunogenicity Study

Female 6–8‐week‐old C57BL/6 mice were subcutaneously vaccinated with 20 µg of mRNA‐LNP on days 0, 3, 7, and 14 and 100 µg of peptides with 50 µg of poly (I:C) (Sigma‐Aldrich) on days 0 and 7. The vaccinated mice were euthanized 7 days after the last vaccination, and splenocytes were harvested to confirm immunogenicity using ELISpot and flow cytometric analysis.

### In Vivo Tumor Challenge Study

Tumor‐bearing mice were subcutaneously vaccinated with 10 to 20 µg of mRNA‐LNPs. ICIs, such as anti‐mouse PD‐1 antibody (*InVivo*MAb, Clone: RMP1‐14, BioXcell, NH, Lebanon) and anti‐mouse Tim‐3 antibody (*InVivo*MAb, Clone: B8.2C12, BioXcell), were intraperitoneally administered at a dose of 200 µg per injection on days 3, 8, and 14 after tumor inoculation. Tumor sizes were measured using an electronic vernier caliper at 2‐ to 4‐day intervals, and tumor volume was calculated using the equation V (mm^3^) = (length × width^2^) ÷ 2.

### TIL Isolation

Tumors were harvested, cut into pieces of appropriate size, and chopped with sterile scissors. The chopped tissue was digested in serum‐free RPMI containing 1 mg mL^−1^ Collagenase D (Sigma‐Aldrich) and 0.1 mg mL^−1^ DNase I (Sigma‐Aldrich) at 37 °C for 1 h. Subsequently, they were grounded through a 70 µm cell strainer and centrifuged at 1000 rpm for 5 min. The supernatant was removed, and 1 mL of ACK lysis buffer was added to the pellet. The ACK lysis buffer was neutralized with RPMI1640 containing 10% fetal bovine serum and centrifuged. The pellet was resuspended in RPMI1640 medium.

### Enzyme‐Linked Immunospot Assay

Mice treated with PBS, peptides, irrelevant mRNA, and mRNA vaccines were euthanized, and splenocytes and tumor lymphocytes were isolated. Subsequently, 2.0 or 5.0 × 10^5^ cells were seeded into each well of anti‐IFN‐γ coated 96‐well plates (Cat.MSIPS4510, Millipore, Burlington, MA, USA) and stimulated with MHC‐I or MHC‐II predicted peptides for 24–48 h in a humidified incubator at 37 °C, 5% CO_2_. After washing the plates, an ELISpot assay was performed following the manufacturer's protocol (Cat. 3321–4APT, Mabtech, Nacka Strand, Sweden). The spots were counted using an ELISpot reader (ImmunoSpot Software, C.T.L corporation, Beaverton, OR, USA). The immunogenicity of neoantigens was evaluated by performing IFN‐γ ELISpot analysis repeatedly.

### In Vitro Cytotoxic T Lymphocyte (CTL) Assay

MC38 cells expressing firefly luciferase (MC38‐Luc) were seeded into 96‐well plates at a density of 5 × 10^4^ cells per well. These cells were then treated with TILs obtained from the tumor tissues of mice treated with PBS or the mRNA vaccine. The control wells were treated with RPMI 1640 medium containing 10% fetal bovine serum and 1% penicillin/streptomycin. The ratio of TILs (Effector cells) and MC38‐Luc cells (Target cells) was 5:1, and the co‐culture was incubated for 18 h at 37 °C in an atmosphere containing 5% CO_2_. After incubation, the cells were washed twice with PBS. Luminescence was measured using a Luciferase Assay System (E4550, Promega Corp., Madison, WI, USA), following the manufacturer's instructions.

### Histology and Immunohistochemistry

Tumor tissues were sectioned into appropriately sized pieces and fixed overnight in a 4% paraformaldehyde solution at room temperature for 24 h. They were then transferred to 70% ethanol before the paraffin‐embedding process. Tissue blocks were sectioned to a thickness of 10 µm, and the sections were mounted on glass slides. H&E staining for pathological analysis was performed by KP&T Technology in South Korea.

IHC for CD8 was performed by staining cells with an anti‐mouse CD8 antibody (Clone EPR21769, Abcam, Cambridge, UK). Following the manufacturer's instructions (HRP/DAB detection IHC kit, ab64264, Abcam), the binding of the CD8 antibody was visualized using 3,3′‐diaminobenzidene (DAB) tetrahydrochloride hydrate.

### Flow Cytometry Analysis

Splenocytes and TILs were seeded into 96‐well plates at densities of 2 × 10^5^ or 1 × 10^6^ cells per well and treated with Fc block (Cat. 553 141, BD Biosciences) to block non‐specific Fc receptor‐mediated fluorescent antibody binding after washing with wash buffer (0.5% fetal bovine serum in PBS). For surface marker analysis, the cells were stained with fluorescence‐labeled mouse antibodies against cell surface markers (including anti‐CD45, anti‐CD4, anti‐CD8, anti‐PD‐1, anti‐CTLA‐4, and anti‐Tim‐3) for 30 min at 4 °C. To assess antigen‐specific cytokine secretion, the cells were stimulated with 200–500 ng/well MHC‐I and MHC‐II peptides at 37 °C in an atmosphere containing 5% CO_2_ for 12 h and stained using the same immune cell population analysis protocol. Subsequently, the cells were permeabilized using Cytofix/Cytoperm (Cat. 554 714, BD Biosciences) and stained with fluorescence‐labeled mouse anti‐IFN‐γ and anti‐TNF‐α antibodies for 30 min at 4 °C. For transcription factor analysis, such as FoxP3, the cells were permeabilized using the FoxP3/Transcription Factor Staining Buffer Set (Cat. 00‐5523‐00, eBioscience) and stained with fluorescence‐labeled mouse anti‐FoxP3 antibodies for 30 min at 4 °C. Flow cytometry and data analysis were performed using a Beckman CytoFLEX (Beckman Coulter, Brea, CA, USA).

### Statistics

Statistical analysis of data was performed using GraphPad Prism (version 9.0; GraphPad, La Jolla, CA, USA) with significance defined as non‐significant (ns; *p* > 0.05) or significant (*p* ≤ 0.05 [*], *p* ≤ 0.01 [**], and *p* ≤ 0.001 [***]). All results from independent experiments are presented as the mean ± standard error of the mean (SEM), unless otherwise stated. Tumor growth was determined using a two‐way analysis of variance, followed by post hoc testing with Dunnett–Turkey's multiple comparison test. The survival rate was determined using the log‐rank test.

## Conflict of Interest

The authors declare no conflict of interest.

## Author contributions

S.C. W.K., H.Y., and J.L. contributed equally to this work. S.C., W.K., H.Y., J.L. wrote, reviewed, and edited the manuscript, wrote original draft, performed visualization, investigation, formal analysis, data curation; S.L., H.J.P., S.J., Y.S.L., Y.J.S., Y.C., S.H.B., and S.Y. wrote, reviewed, and edited the manuscript, performed visualization, validation, software, methodology, investigation, formal analysis, data curation; G.R., D.H., A.O., E.J.C., S.Y.L., H.C., J.K., Y.L., and S.L. performed methodology, investigation, formal analysis, data curation; S.I.P., D.K.K., J.C., K.T.K., and K.K. wrote, reviewed, and edited the manuscript, performed software, methodology, investigation; J.H.N. wrote, reviewed, and edited the manuscript, wrote original draft, performed visualization, supervision, resources, project administration, methodology, investigation, funding acquisition, conceptualization.

## Supporting information



Supporting Information

## Data Availability

The data that support the findings of this study are available from the corresponding author upon reasonable request.
