# Supplementary material for: Rapid‐Turnaround Co‐Administration of mRNA‐Based MHC‐I and MHC‐II‐Restricted Neoantigens Enhances Immune Responses of Antigen‐Specific CD8+ T Cells and Anti‐Cancer Efficacy in Colorectal Cancer
Source: Adv Sci (Weinh). 2025 Jul 23;12(39):e06426. doi: 10.1002/advs.202506426 (PMC12533368; doi:10.1002/advs.202506426)
Supplement: Supplementary file 1 — Supporting Information [file ADVS-12-e06426-s001.docx]

Supporting Information

**Rapid-Turnaround Co-Administration of mRNA-Based MHC-I and MHC-II-Restricted Neoantigens Enhances Immune Responses of Antigen-Specific CD8^+^ T Cells and Anti-Cancer Efficacy in Colorectal Cancer**

*Seongje Cho, Woori Kwak, Hyunho Yoon, Jisun Lee, Seonghyun Lee, Hyo‐Jung Park, Sohee Jo, Yu-Sun Lee, Yu-Jeong Seo, Youngran Cho, Seo-Hyeon Bae, Subin Yoon, Gahyun Roh, Dahyeon Ha, Ayoung Oh, Eun-Jin Choi, Soo-Yeon Lee, Huijeong Choi, Jungmin Kim, Yeeun Lee, Sowon Lee, Sang-In Park, Dae-Keun Kim, Jun Chang, Ki Tae Kim, Kwoneel Kim, and Jae-Hwan Nam**

**Table S1.** Number of identified genomic variants using two variant callers.

|  | HaplotypeCaller | Samtools |
| --- | --- | --- |
| SNVs^a)^ | 51,543 | 226,026 |
| InDels^b)^ | 13,959 | 37,829 |
| SNVs (Filtered using Vcftools) | 32,703 | 21,888 |
| InDels (Filter using Vcftools) | 9,116 | 3,337 |
| Tumor Specific SNVs | 6,638 | 673 |
| Tumor Specific InDels | 1,088 | 136 |
| Consensus SNVs | 546 | |
| Consensus InDels | 68 | |

^a)^SNVs, single nucleotide variants; ^b)^InDels, insertions/deletions

**Table S2.** Information of MHC-I-restricted neoantigen candidates.

| **MHC**  **Class** | **No** | **Epitope** | **Wild A.A.**^a)^ | **Mutated A.A.** | **Source** | **Target Gene** |
| --- | --- | --- | --- | --- | --- | --- |
| MHC-I | CNM-I-01 | SYARR **\|**^b)^ DPSFSSL | DAFTDSA | DPSFSSL | Gene Fusion | *Atxn2_*  *Gm26647* |
|  | CNM-I-02 | RGDDIERLL | MLKDEVRTL | RGDDIERLL | Gene Fusion | *Etv6_*  *Prmt8*^c)^ |
|  | CNM-I-03 | FYRGKQ **\|** LDELV | LENGY | LDELV | Gene Fusion | *Uhrf2_*  *Unc93b1* |
|  | CNM-I-04 | **[S]**^d)^ANG**[F]**SK**[V][D]**TL | PANGCSKQTL | S,F,V,D | SNV^e)^/InDel^f)^ | *Sp110* |
|  | CNM-I-05 | S**[S]**F**[L]**YSS**[L]** | SIFRYSSC | S,L,L | SNV/InDel | *Hace1* |
|  | CNM-I-06 | S **\|** QQSPSPSL | GKEHNGVR | QQSPSPSL | SNV/InDel | *Bcorl1*^c)^ |
|  | CNM-I-07 | IHPKA**[F]**PLI | L | F | SNV/InDel | *Hp1bp3* |
|  | CNM-I-08 | YG**[F]**PGILEFF | S | F | SNV/InDel | *Cyfip2* |
|  | CNM-I-09 | SG**[E]**NNFQLV | D | E | SNV/InDel | *Gm11757* |
|  | CNM-I-10 | RNNSFGVS**[W]** | C | W | SNV/InDel | *Alms1* |
|  | CNM-I-11 | Q**[V]**YTAFQAA | G | V | SNV/InDel | *Cnppd1* |
|  | CNM-I-12 | **[S][G]**C**[F]**RRQL | EECRRQL | S,G,F | SNV/InDel | *Cep78* |
|  | CNM-I-13 | SYALILRT**[I]**L | V | I | SNV/InDel | *Olfr577* |
|  | CNM-I-14 | KHA**[S]**VIDNL | R | S | SNV/InDel | *Taar2* |
|  | CNM-I-15 | EYHMVHL**[L]** | V | L | SNV/InDel | *Herpud2* |
|  | CNM-I-16 | **[D]**SPGRLHF | G | D | SNV/InDel | *Akap3* |
|  | CNM-I-17 | LGPDD**[P]**VEAVL | L | P | SNV/InDel | *Fras1* |
|  | CNM-I-18 | L **\|** GPFPNSL | VLPSNS | GPFPNS | Gene Fusion | *Cenpc1_Tmprss11* |
|  | CNM-I-19 | RLFYRGKQ **\|** L | VPDGPEAP | RLFYRGKQ | Gene Fusion | *Uhrf2_*  *Unc93b1* |
|  | CNM-I-20 | RGKQ **\|** LDEL | LENG | LDEL | Gene Fusion | *Uhrf2_*  *Unc93b1* |

^a)^A.A., amino acids; ^b)^**|**, gene fusion; ^c)^Frameshift mutation; ^d)^**[Bold]**, mutated amino acids; ^e)^SNV, single nucleotide variants; ^f)^InDel, insertions/deletions.

A.A., Amino acids; SNV, single nucleotide variants; InDel, insertions/deletions; [Bold], mutated amino acids; |, gene fusion; **,* Frameshift mutation

**Table S3.** Information of MHC-II-restricted neoantigen candidates.

| **MHC**  **Class** | **No** | **Epitope** | **Wild A.A.**^a)^ | **Mutated A.A.** | **Source** | **Target Gene** |
| --- | --- | --- | --- | --- | --- | --- |
| MHC-II | CNM-II-01 | LRKL**[L]**^b)^SKALRVATATPPAAPHSSLKTSS | V | L | SNV^c)^/InDel^d)^ | *Cnppd1* |
|  | CNM-II-02 | AALMLATRHT**[D]**LIRAATPPATMCPLSEE | N | D | SNV/InDel | *Cnppd1* |
|  | CNM-II-03 | LSKEQER**[A]**ALMLATRHTDLIRAATPPAT | P | A | SNV/InDel | *Cnppd1* |
|  | CNM-II-04 | NSSWSFQAPPPL**[R]**VKGPLPSYPNSQLAL | P | R | SNV/InDel | *Sp110* |
|  | CNM-II-05 | MKVISAHAGSQPA**[T]**PIPPSTPPPLPCAV | S | T | SNV/InDel | *Sp110* |
|  | CNM-II-06 | GYTHTIFW**[S]**YGYVCLSLFWLSTSPLALR | R | S | SNV/InDel | *Hace1* |
|  | CNM-II-07 | LK**[Q]**TPRRHSDYTAVMEALQAMKAVCSNI | R | Q | SNV/InDel | *Prex2* |
|  | CNM-II-08 | LFGMDLN**[R]**KTHRRMAALRVTKPYLDIGC | K | R | SNV/InDel | *Nlrp12* |
|  | CNM-II-09 | IQGKKGVYLT**[D]**IMPQGVAMKAGVLADDH | N | D | SNV/InDel | *Pdzk1* |
|  | CNM-II-10 | FSYALILRT**[I]**LSIASRAERLKALNTCVS | V | I | SNV/InDel | *Olfr577* |
|  | CNM-II-11 | PQTPRIVRQISISKISALQFSQEPASD**[R]** | P | R | SNV/InDel | *Rab44* |
|  | CNM-II-12 | S **\|**^e)^ GEQRPPSAPSACH  SSQACAMCPPCIHT | Frameshift | | Gene Fusion | *Etv6_*  *Prmt8*^f)^ |

^a)^A.A., amino acids; ^b)^**[Bold]**, mutated amino acids; ^c)^SNV, single nucleotide variants; ^d)^InDel, insertions/deletions; ^e)^SNV, single nucleotide variants; ^f)^InDel, insertions/deletions.

**Table S4.** Information of MHC-I-restricted short and long antigen sequence.

| **MHC**  **Class** | **No** | **Description** | **Sequence** | **Length of amino acids** |
| --- | --- | --- | --- | --- |
| MHC-I | CNM-I-08 | Short antigen | YGF^a)^PGILEFF | 10 aa |
|  |  | Long antigen | MPKICRLPRHEYG**F**PGILEFFHHQLKD | 27 aa |
|  | CNM-I-13 | Short antigen | SYAL**I**LRTIL | 10 aa |
|  |  | Long antigen | GVDSLLILFSYAL**I**LRTILSIASRAER | 27 aa |
|  | CNM-I-19 | Short antigen | **RLFYRGKQ \|**^b)^L | 9 aa |
|  |  | Long antigen | VRPECQ**RLFYRGKQ \|** LDELVG  AYPNYNE | 27 aa |

^a)^Red alphabet, mutated amino acid; ^b)^|, gene fusion.

**Table S5.** The minimal epitopes of MHC-I- or MHC-II-restricted neoantigens.

| **MHC**  **Class** | **No** | **Sequence** | **Length of amino acids** |
| --- | --- | --- | --- |
| MHC-I | CNM-I-13-01 | LLILFSYAL**I**^a)^ | 10 aa |
|  | CNM-I-13-02 | LILFSYAL**I**L | 10 aa |
|  | CNM-I-13-03 | ILFSYAL**I**LR | 10 aa |
|  | CNM-I-13-04 | LFSYAL**I**LRT | 10 aa |
|  | CNM-I-13-05 | FSYAL**I**LRTI | 10 aa |
|  | CNM-I-13-06 | SYAL**I**LRTIL | 10 aa |
|  | CNM-I-13-07 | YAL**I**LRTILS | 10 aa |
|  | CNM-I-13-08 | AL**I**LRTILSI | 10 aa |
|  | CNM-I-13-09 | L**I**LRTILSIA | 10 aa |
|  | CNM-I-13-10 | **I**LRTILSIAS | 10 aa |
| MHC-II | CNM-II-04-01 | NSSWSFQAPPPL**R**V | 14 aa |
|  | CNM-II-04-02 | SSWSFQAPPPL**R**VK | 14 aa |
|  | CNM-II-04-03 | SWSFQAPPPL**R**VKG | 14 aa |
|  | CNM-II-04-04 | WSFQAPPPL**R**VKGP | 14 aa |
|  | CNM-II-04-05 | SFQAPPPL**R**VKGPL | 14 aa |
|  | CNM-II-04-06 | FQAPPPL**R**VKGPLP | 14 aa |
|  | CNM-II-04-07 | QAPPPL**R**VKGPLPS | 14 aa |
|  | CNM-II-04-08 | APPPL**R**VKGPLPSY | 14 aa |
|  | CNM-II-04-09 | PPPL**R**VKGPLPSYP | 14 aa |
|  | CNM-II-04-10 | PPL**R**VKGPLPSYPN | 14 aa |
|  | CNM-II-04-11 | PL**R**VKGPLPSYPNS | 14 aa |
|  | CNM-II-04-12 | L**R**VKGPLPSYPNSQ | 14 aa |
|  | CNM-II-04-13 | **R**VKGPLPSYPNSQL | 14 aa |

^a)^Red alphabet, mutated amino acid


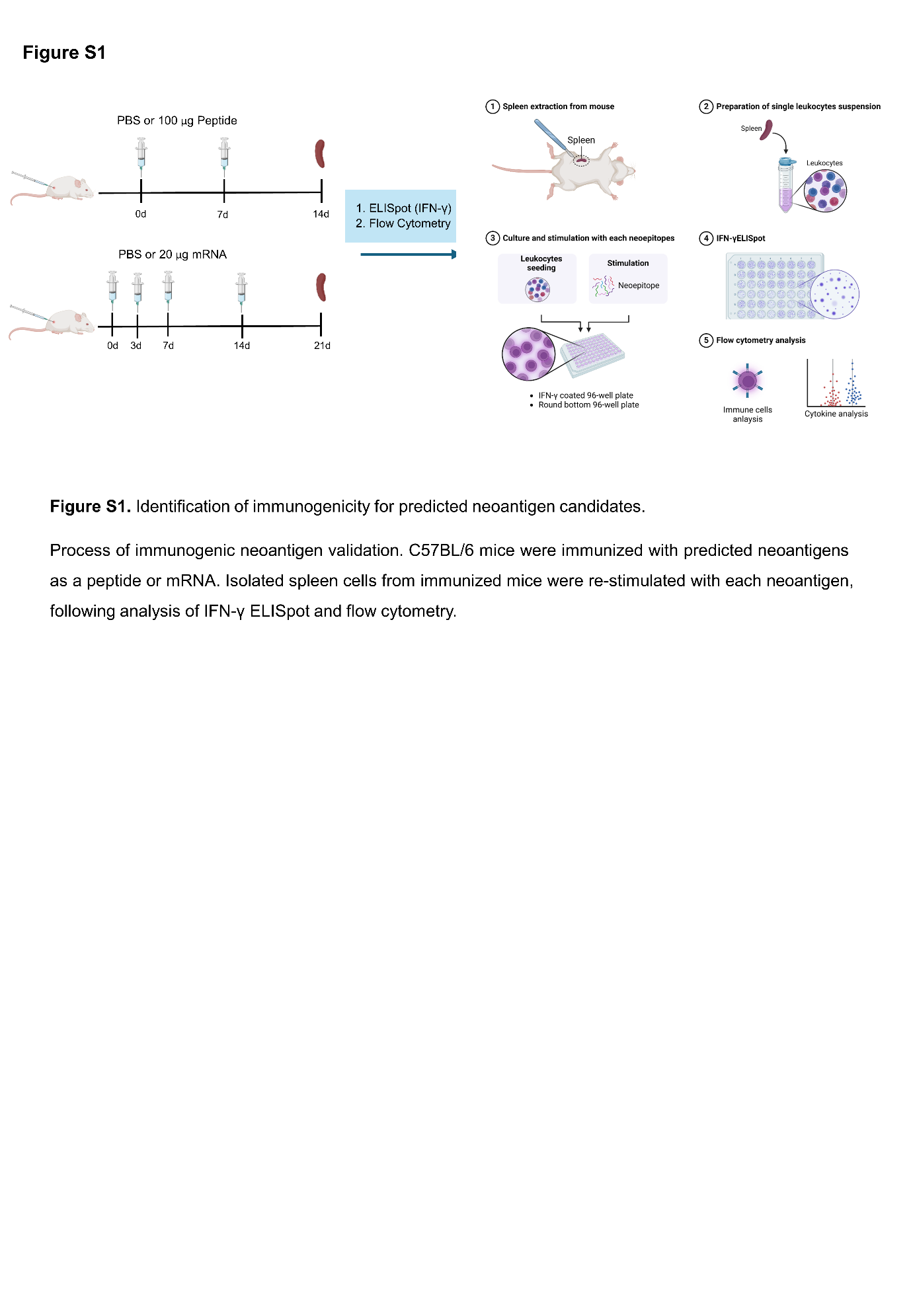


**Figure S1.** Identification of immunogenicity for predicted neoantigen candidates. Process of immunogenic neoantigen validation. C57BL/6 mice were immunized with predicted neoantigens as a peptide or mRNA. Spleen cells isolated from immunized mice were re-stimulated with each neoantigen and analyzed using IFN-γ ELISpot and flow cytometry


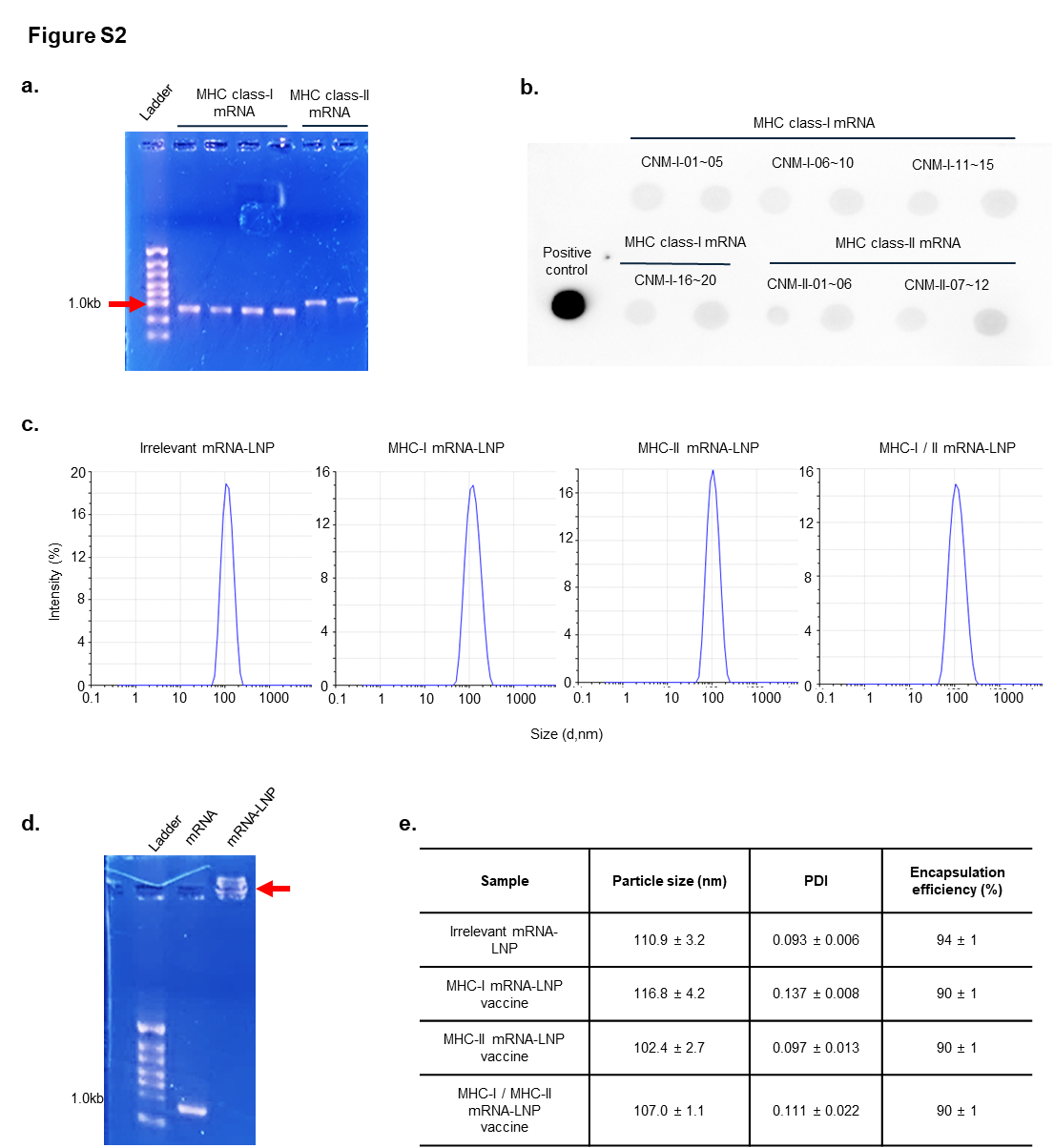


**Figure S2.** Characterization of mRNA and mRNA-LNP encoding neoantigens. **a.** Agarose gel electrophoresis of MHC-I or MHC-II neoantigen-encoding mRNAs. The arrow indicates 1.0 kb of ssRNA in standard size marker. **b.** Dot blot analysis for double stranded RNA impurities. MHC-I/II mRNA vaccines were analyzed in dot blot with J2 dsRNA-specific mAb. **c.** Physicochemical characteristics of mRNA-LNPs as measured by dynamic light scattering (DLS). **d.** Agarose gel electrophoresis of naked mRNA or mRNA-LNP for encapsulation efficiency of mRNA. The arrow indicates mRNA-LNP complex. **e.** Particle size, polydispersity index (PDI), and encapsulation efficiency of the produced LNPs. The irrelevant mRNA encoded the sequences of GFP (Green Fluorescent Protein). Data presented as mean ± standard deviation (SD).


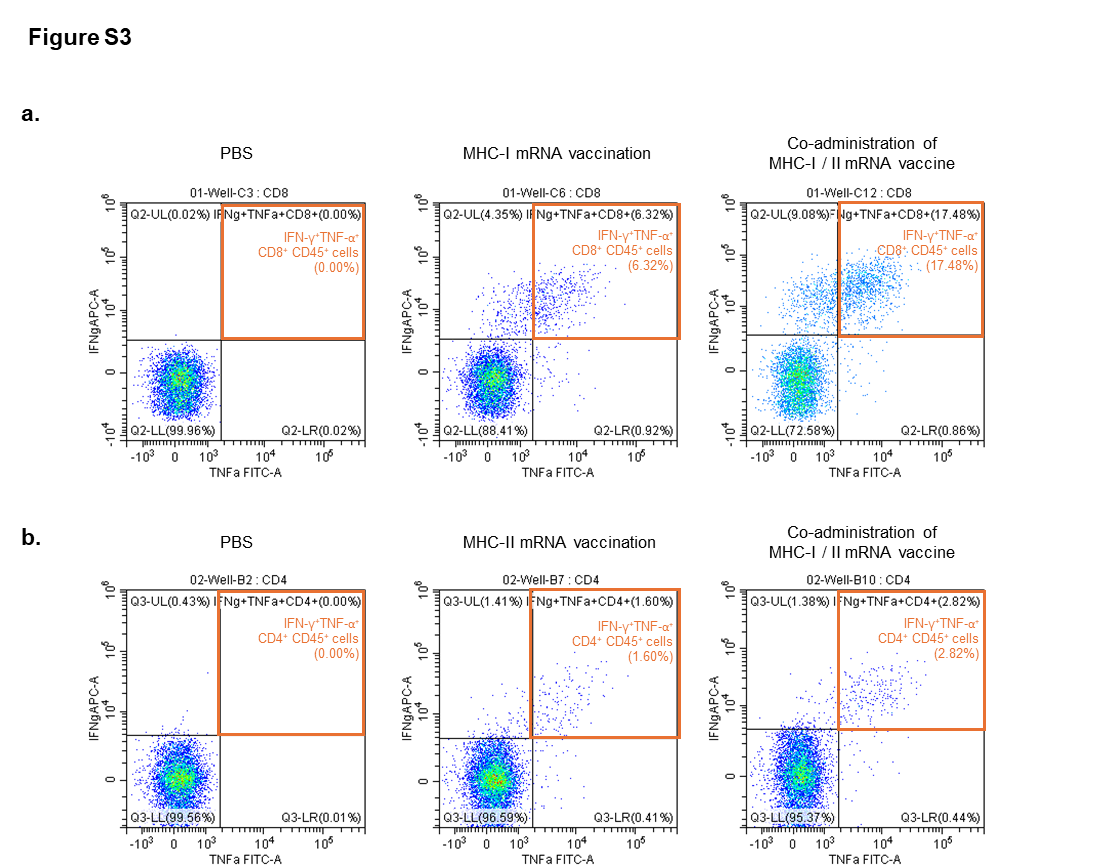


**Figure S3.** Co-administration of MHC-I and MHC-II neoantigens significantly increased secretion of cytokines by antigen-specific T cell responses. **a.** Representative data of cytokine secretion of CD8^+^ T cells by MHC-I minimal epitopes. **b.** Representative data of cytokine secretion of CD4^+^ T cells by MHC-II minimal epitopes. To investigate the antigen-specific secretion of cytokines in T cells, such as IFN-γ^+^/TNF-α^+^ CD4^+^ T cells, IFN-γ^+^/TNF-α^+^ CD8^+^ T cells, the splenocytes isolated from vaccinated mice were analyzed using a flow cytometric analysis after stimulation with each peptide (CNM-I-13-06 or CNM-II-04-10). The orange boxes indicate the population of IFN-γ^+^TNF-α^+^ T cells in spleen cells immunized with indicated vaccines.


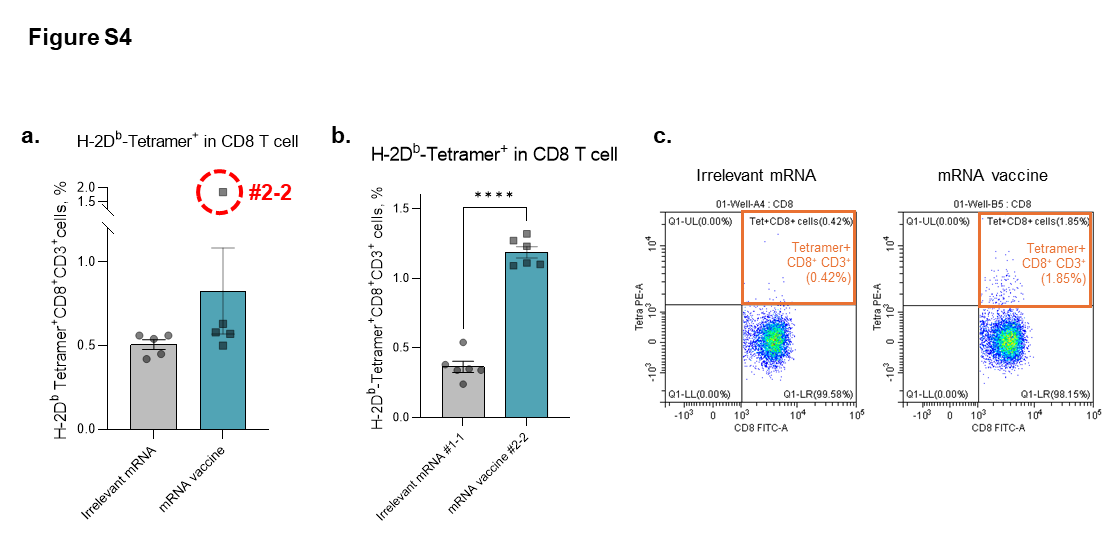


**Figure S4.** MHC-I antigen-specific T cell detection using H-2D^b^ tetramer. **a.** The spleen cells were collected from immunized mice (n=5) and stained with PE-H-2D^b^ tetramer conjugated with CNM-13-06 (10-mer) to detect MHC-I antigen-specific T cells using flow cytometry. The red dotted circle indicates the individual mouse (No: #2-2) that increased the population of PE-H-2D^b^ tetramer^+^ of CD8^+^ T cells. **b.** Flow cytometric analysis for 6 replicates test in individual mice (mouse No#1-1 was administered Irrelevant mRNA and mouse No#2-2 MHC-I/II mRNA vaccine). **c.** Representative data of flow cytometric analysis for detection of MHC-I antigen-specific T cells using H-2D^b^ tetramer conjugated with CNM-13-06. Data of flow cytometric analysis are shown as the mean ± SEM analyzed using an unpaired two-tailed Student’s t-test (****p < 0.0001, ***p < 0.001, **p < 0.01, *p < 0.05).


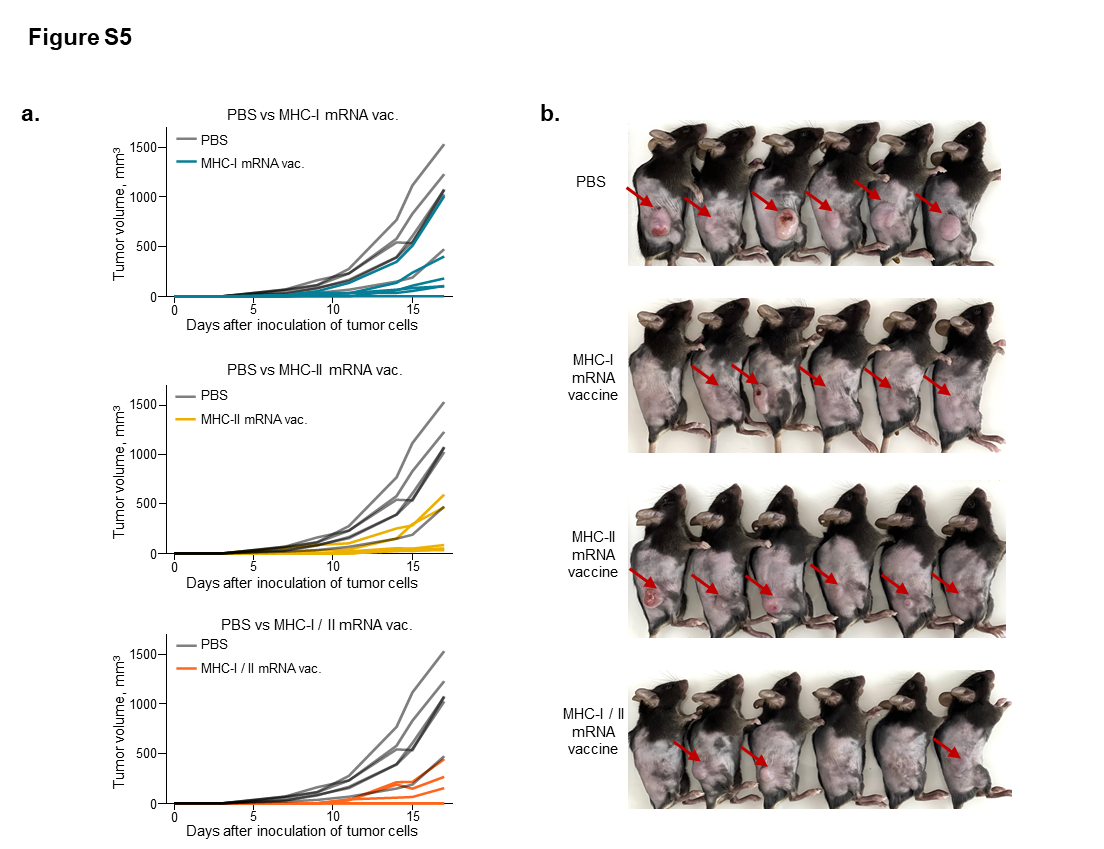


**Figure S5.** Co-administration of MHC-I and MHC-II mRNA vaccines increase anti-cancer efficacy in a colon cancer murine model. **a.** Tumor volume of individual mice treated with PBS, MHC-I, MHC-II vaccine or both vaccines at indicated time points (n=6). **b.** Tumor images of mice immunized with MHC-I, MHC-II, or both vaccines at 17 days after tumor inoculation.


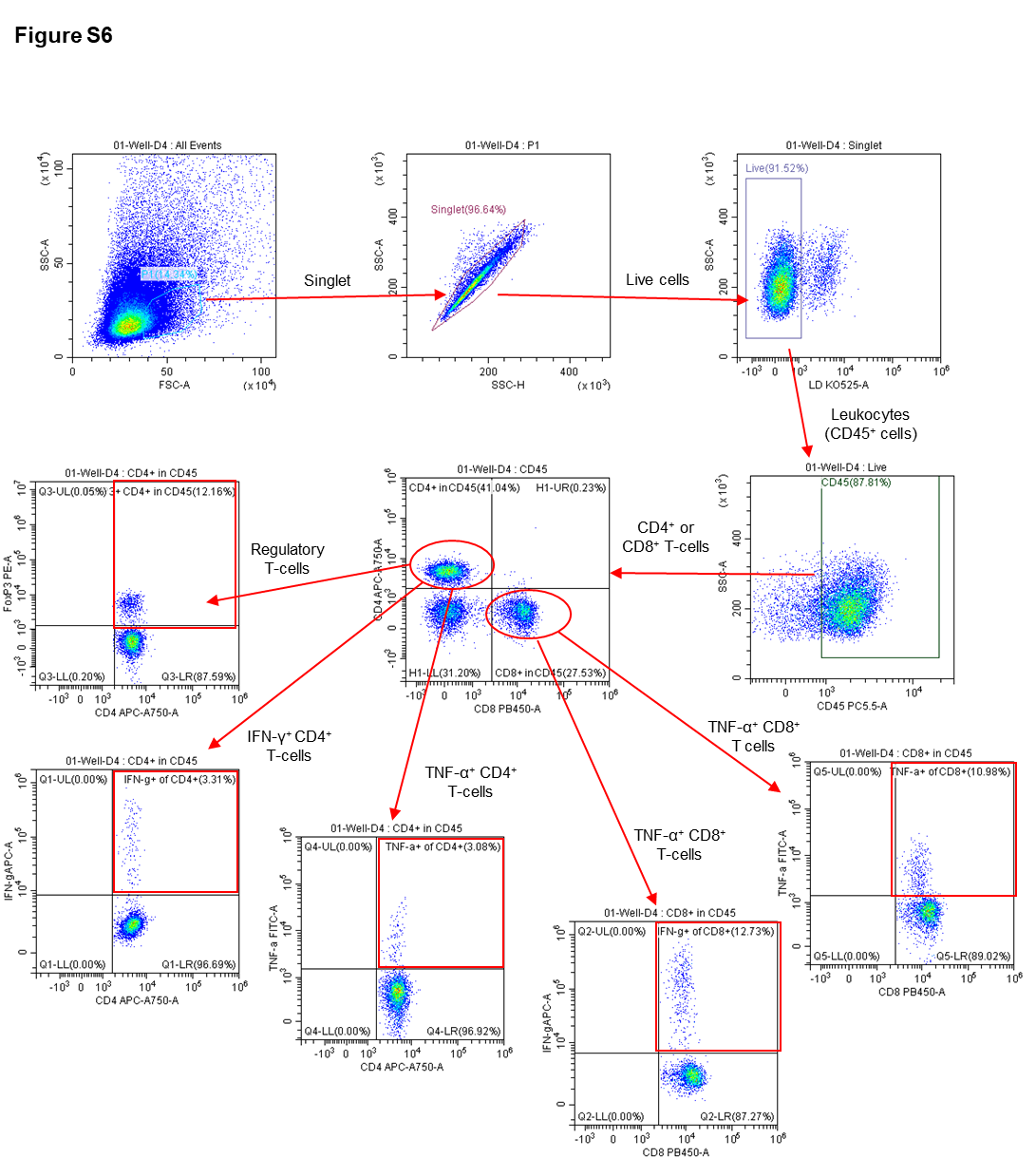


**Figure S6.** Gating strategy for flow cytometry analysis. To investigate the population of immune cells, such as IFN-γ^+/^TNF-α^+^ CD4^+^ T cells, IFN-γ^+/^TNF-α^+^ CD8^+^ T cells, and regulatory T cells, the splenocytes isolated from vaccinated mice were analyzed using a flow cytometry method. Lymphocytes, including single cells (singlets), were selected on a SSC-A versus SSC-H plot to exclude signaling data from doublets. Then, CD4^+^ cells or CD8^+^ cells are selected from live CD45^+^ cells. The population of regulatory T cells was selected FoxP3^+^CD4^+^CD45 ^+^ cells. To confirm the secretion of cytokines by tumor-specific neoantigens, the cells were stained with fluorescence-labeled anti-mouse IFN-γ and TNF-α antibodies.


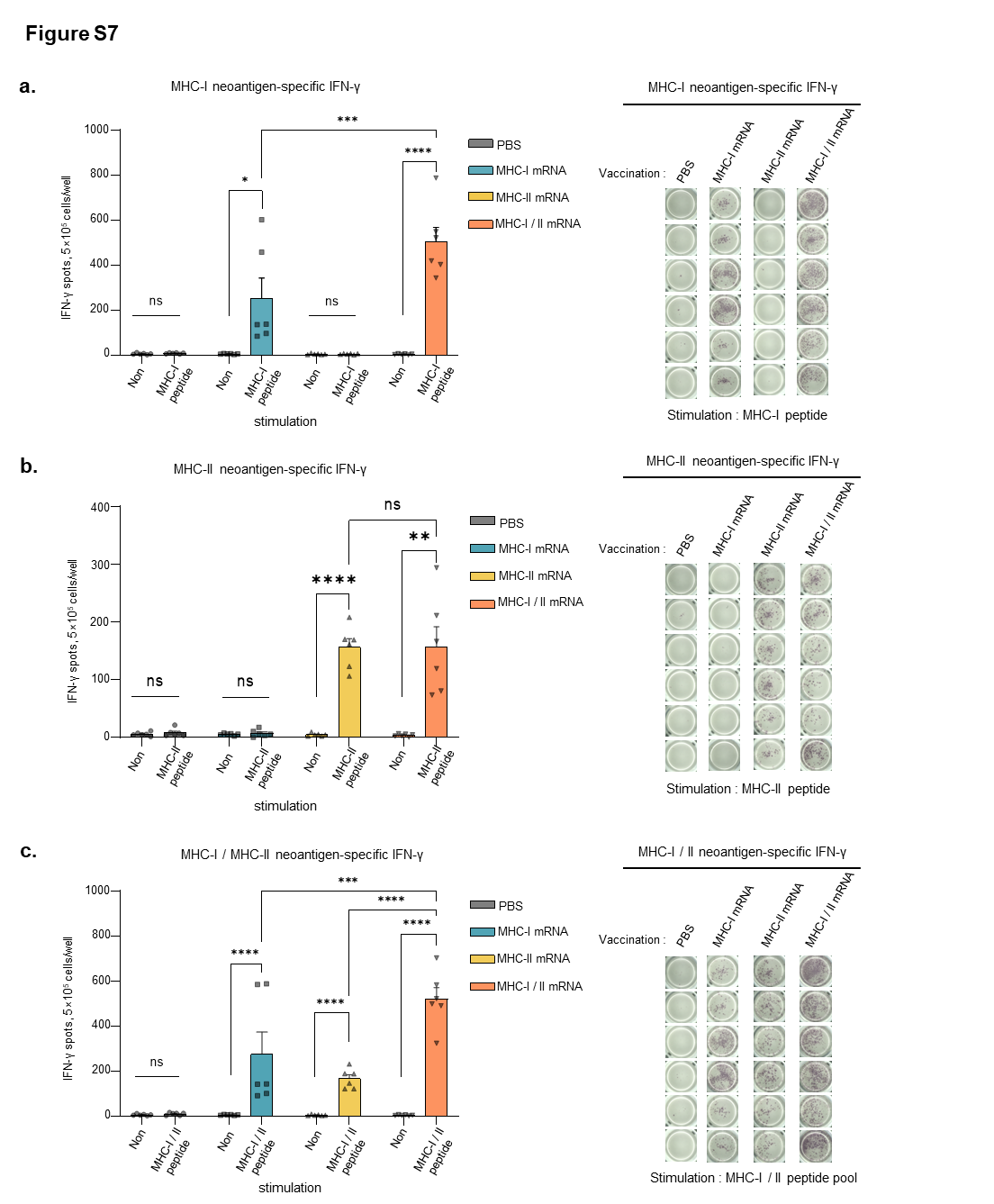


**Figure S7.** Neoantigen-specific immune response by vaccination in a colon cancer murine model. **a−c.** MC38 tumor-bearing mice were vaccinated with PBS, MHC-I mRNA vaccine, MHC-II mRNA vaccine, or both (co-administration). For IFN-γ ELISpot analysis, the spleen cells re-stimulated with MHC-I peptides (pooled with CNM-I-08, CNM-I-13, and CNM-I-19) (**a**), MHC-II peptides (pooled CNM-II-01, and CNM-II-04; **b**), or MHC-I/II peptide pool (pooled with CNM-I-08, CNM-I-13, CNM-I-19, CNM-II-01, and CNM-II-04 [**c**]). ELISpot data shown as mean ± standard error of the mean analyzed using an unpaired two-tailed Student’s t-test (****p < 0.0001, ***p < 0.001, **p < 0.01, *p < 0.05).


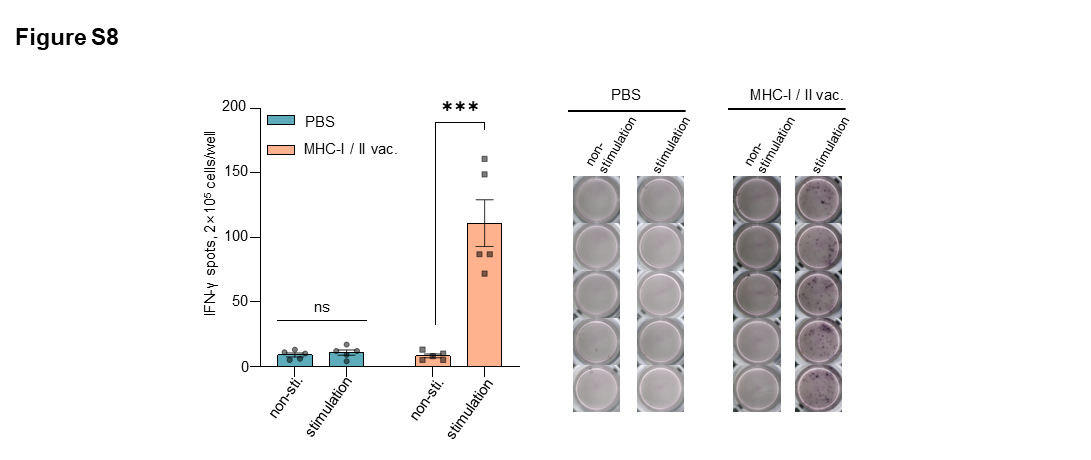


**Figure S8.** Neoantigen-specific immune response according to vaccination in a surgically treated colon cancer mouse model. Mice were subcutaneously inoculated with 2×10^5^ MC38 tumor cells. After 14 days, tumor tissues were surgically removed, and the mice were either administered PBS or co-administered MHC-I and MHC-II vaccines (n=5 per group) on days 6, 9, and 12. For IFN-γ ELISpot analysis, the spleen cells harvested from immunized mice were re-stimulated with MHC-I/II peptides pool (CNM-I-08, CNM-I-13, CNM-I-19, CNM-II-01, and CNM-II-04). ELISpot data are shown as the mean ± standard error of the mean, analyzed unpaired a two-tailed Student’s t-test (****p < 0.0001, ***p < 0.001, **p < 0.01, *p < 0.05).


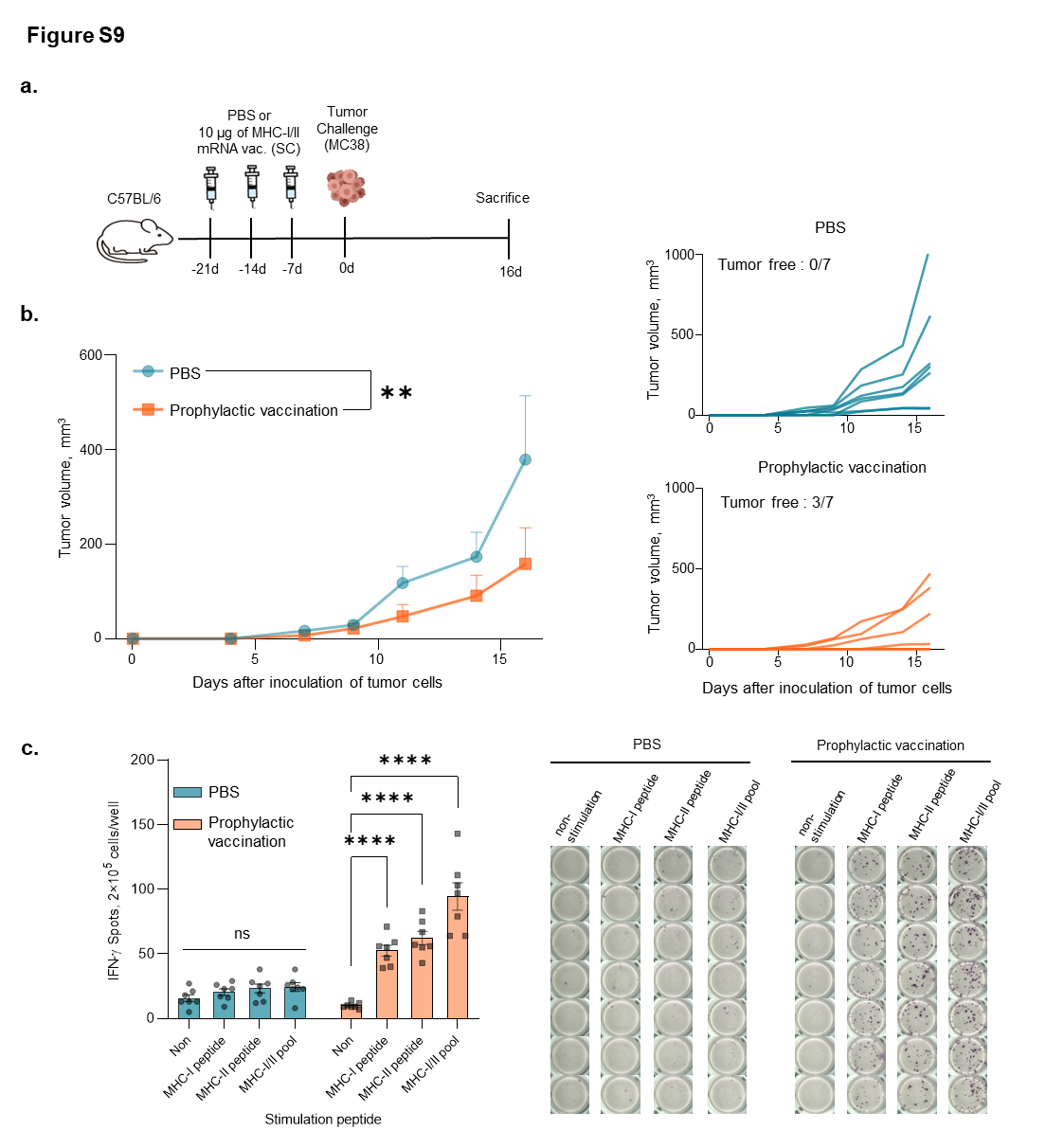


**Figure S9.** Reduction of tumor growth burden by prophylactic vaccination with mRNA-based neoantigens. **a.** Experimental design of prophylactic vaccination study. C57BL/6 mice were immunized with PBS or MHC-I/II vaccines on days -21, -14, and -7 (n=7 per group). Seven days after the last vaccination, mice were subcutaneously inoculated with 1×10^5^ MC38 tumor cells. **b.** Tumor growth was monitored until 17 days after tumor inoculation. **c.** IFN-γ ELISpot analysis of spleen cells re-stimulated with pooled MHC-I/II peptides (CNM-I-08, CNM-I-13, CNM-I-19, CNM-II-01, and CNM-II-04). Tumor growth curves are shown as the mean ± standard error of the mean, analyzed using a two-way ANOVA with Dunnett’s multiple comparisons test. ELISpot data are shown as mean ± standard error of the mean, analyzed using an unpaired two-tailed Student’s t-test (****p < 0.0001, ***p < 0.001, **p < 0.01, *p < 0.05).


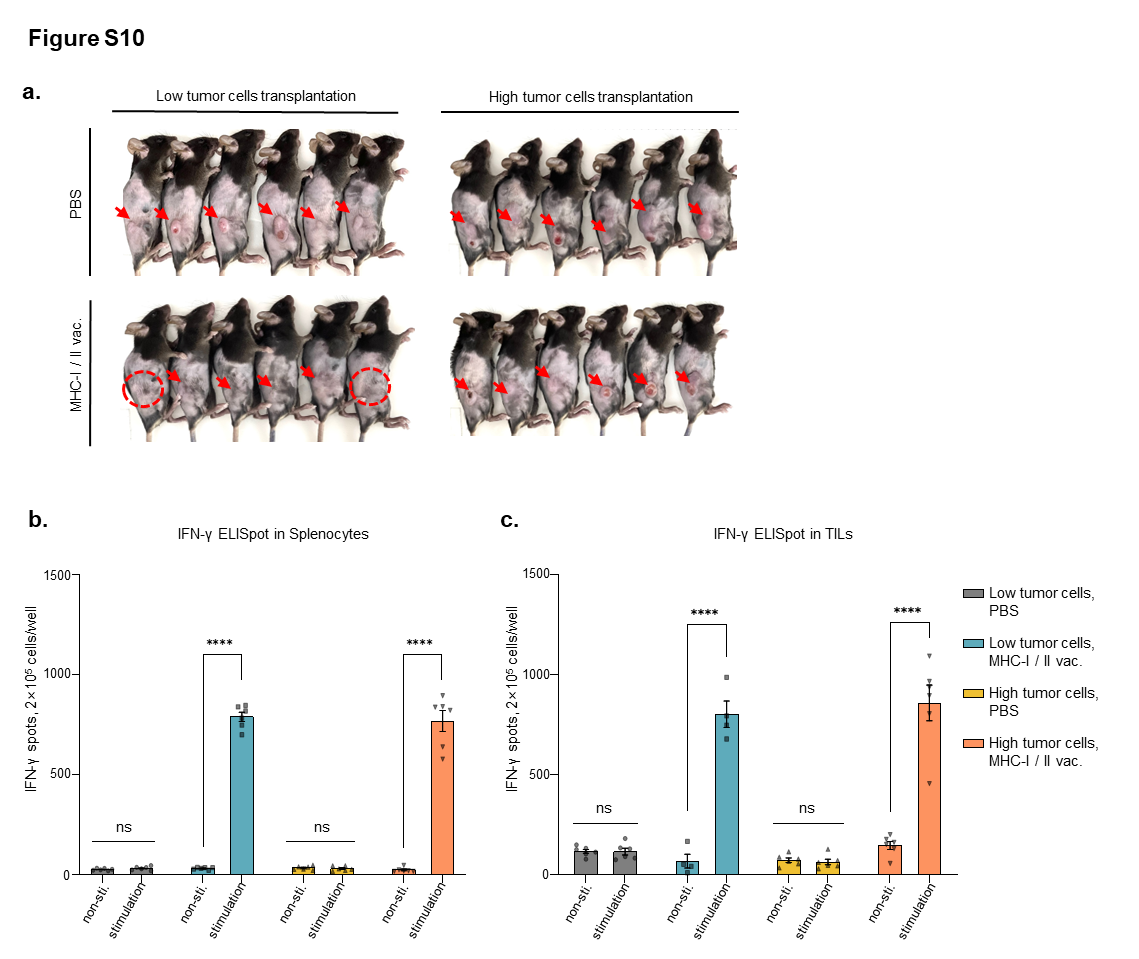


**Figure S10.** Antitumor efficacy depends on the amount of transplanted tumor cells. **a.** Images of tumor-bearing mice according to the amount of cancer cells transplanted. The arrows indicated tumor tissues and the dotted circles are shown tumor free mice. **b.** IFN-γ ELISpot analysis of splenocytes isolated from mice treated with mRNA vaccines (n=6 per group). **c.** IFN-γ ELISpot analysis of TILs isolated from mice treated with mRNA vaccines (n=6 per group). IFN-γ ELISpot data are shown as the mean ± standard error of the mean, analyzed using unpaired two-tailed Student’s t-test (****p < 0.0001, ***p < 0.001, **p < 0.01, *p < 0.05).


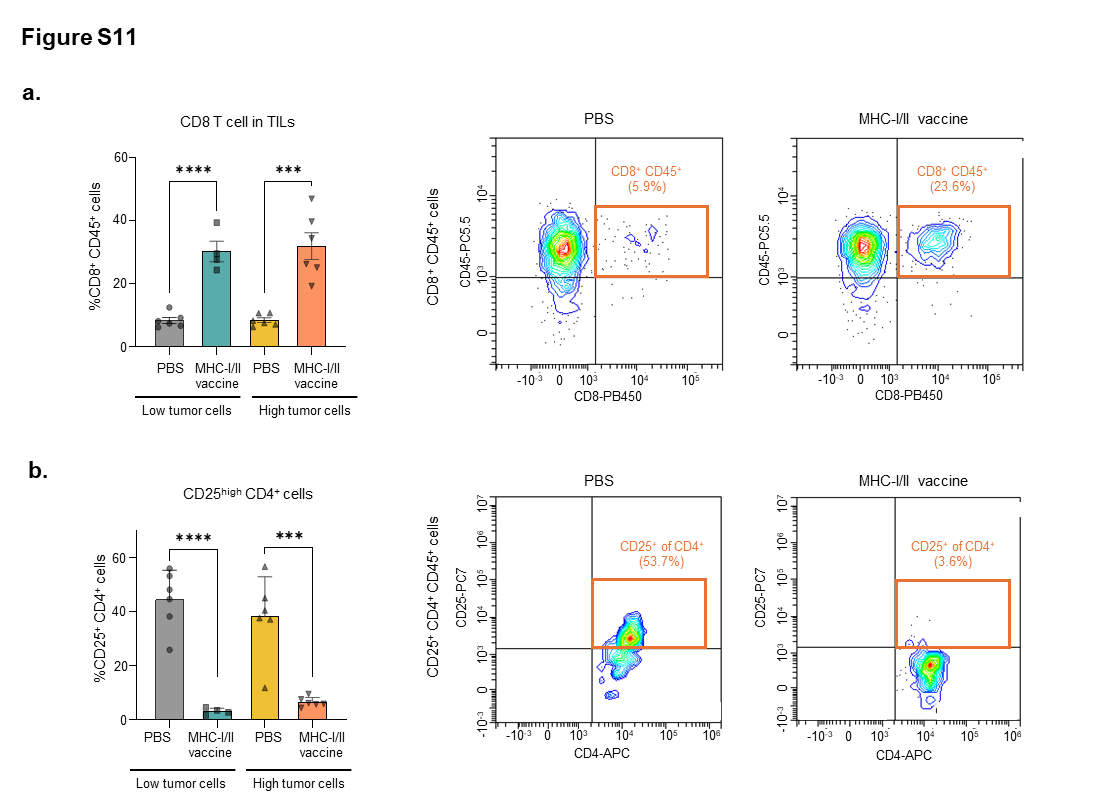


**Figure S11.** The population of cytotoxic T cells and suppressor T cells in TILs isolated from mice with mRNA vaccination. Flow cytometric analysis for T cell population in TILs (n=6 per group). **a.** Population of CD8^+^ T cells (CD8^+^CD45^+^) (left) and representative data of flow cytometry analysis (right). **b.** Population of suppressor T cells (CD25^+^ of CD4 T cells) (left) and representative data of flow cytometry analysis (right). Data from flow cytometric analysis was shown as the mean ± standard error of the mean, analyzed using an unpaired two-tailed Student’s t-test (****p < 0.0001, ***p < 0.001, **p < 0.01, *p < 0.05).

**
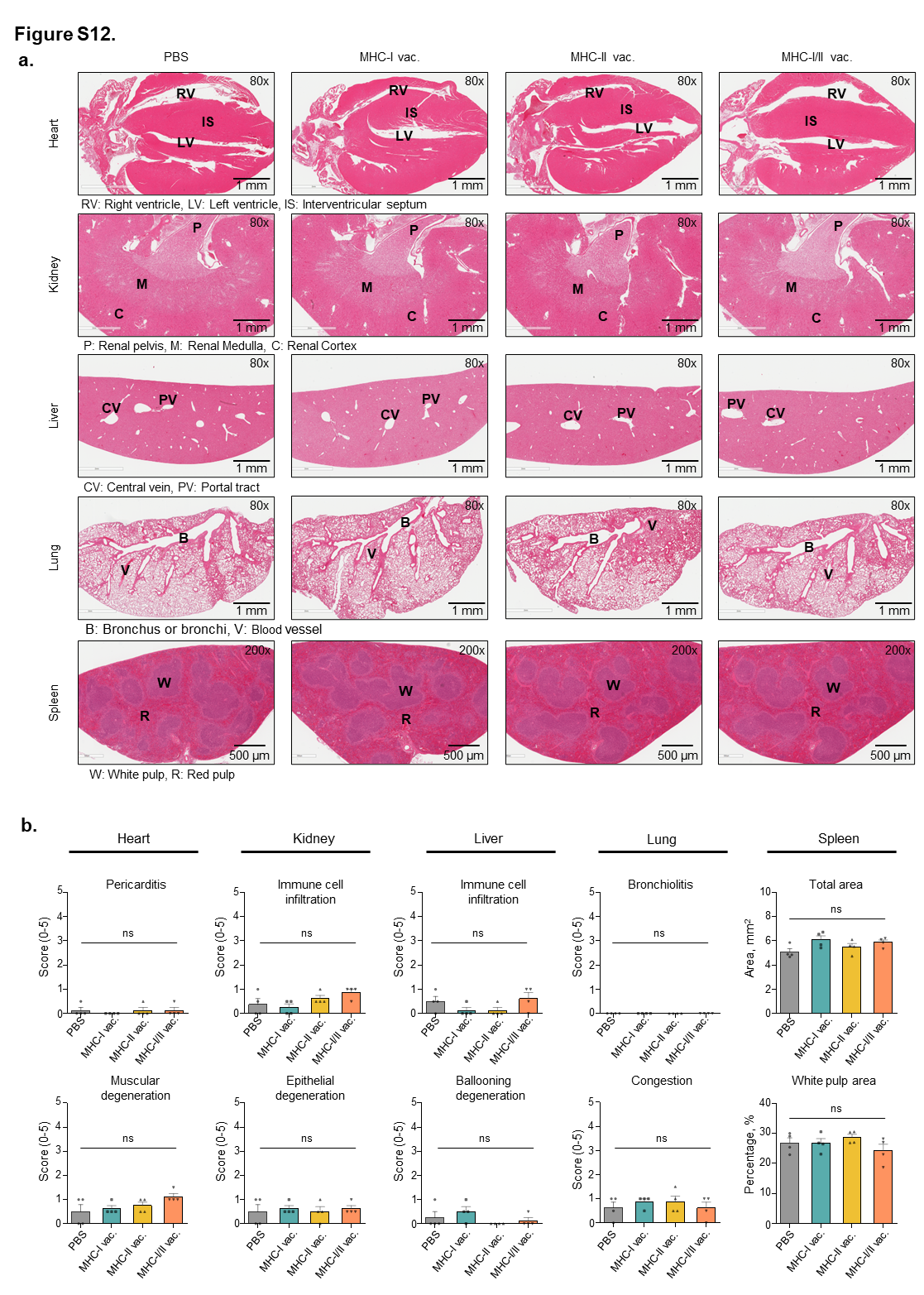
**

**Figure S12.** Representative histopathological safety assessments and image (H&E) of cancer vaccine-treated organs. **a.** Histopathological safety assessment of the mRNA-based cancer vaccine was conducted on the heart, kidney, liver, lung, and spleen. C57Bl/6 mice were immunized with PBS, 20 μg of mRNA-based MHC-I, MHC-II neoantigens, or co-administration on 0, 4, 7, and 14 days (n=4). After 1 week, the tissue sections from mice were stained with hematoxylin and eosin (H&E) and examined under a microscope. **b.** Evaluation of pathological changes, such as inflammation, structural abnormalities, fibrosis, or other pathological findings, were observed in any of the groups, including PBS, MHC-I, MHC-II, and combination vaccine group. Data shown as mean ± standard error of the mean, analyzed using a one-way ANOVA with Dunnett’s multiple comparisons test (****p < 0.0001, ***p < 0.001, **p < 0.01, *p < 0.05).
